# Hydrogenative coupling of nitriles with diamines to benzimidazoles using lignin-derived Rh_2_P catalyst

**DOI:** 10.1016/j.isci.2021.103045

**Published:** 2021-08-28

**Authors:** Jiarui Zhang, Ruxu Yao, Jinzhu Chen, Tao Li, Yisheng Xu

**Affiliations:** 1 State Key Laboratory of Chemical Engineering, International Joint Research Center of Green Energy Chemical Engineering, East China University of Science and Technology, Shanghai 200237, China; 2 College of Chemistry and Materials Science, Guangdong Provincial Key Laboratory of Functional Supramolecular Coordination Materials and Applications, Jinan University, Guangzhou 511443, China

**Keywords:** chemistry, inorganic chemistry, catalysis, organic chemistry, organic synthesis

## Abstract

Nitrile (C≡N bond) activation for direct organic synthesis has been less explored so far due to a high redox potential of nitrile and its low dissociation energy of C−CN bond. Herein, we demonstrate a direct reductive coupling of nitriles and 1,2-phenylenediamines to yield various benzimidazoles in excellent yields (95%–99%) by using rhodium phosphide (Rh_2_P) catalyst supported on lignin-derived carbon (LC) using H_2_ (or hydrazine hydrate) as a hydrogen source. The high catalytic performance of Rh_2_P/LC is attributed to enhanced charge transfer to Rh and strong P−Rh interactions. Our isotope trace experiment confirms the presence of H/D exchange between H_2_ and the inert –CD_3_ group of CD_3_CN *via* an intramolecular D-shift. Reusability of Rh_2_P/LC is further demonstrated by a seven-time recycling without evident loss of activity. This research thus highlights a great potential in organic transformation with nitrile as a synthetic building block.

## Highlights

• Nitrile was developed as synthetic building block for organic synthesis
• Reductive coupling of nitriles to 1,2-phenylenediamines yielded benzimidazoles
• Strong P−Rh interaction and charge transfer to Rh enhanced Rh_2_P activity
• H/D exchange between H_2_ and –CD_3_ in CD_3_CN occurred via intramolecular D-shift

## Graphical abstract

**Figure:**
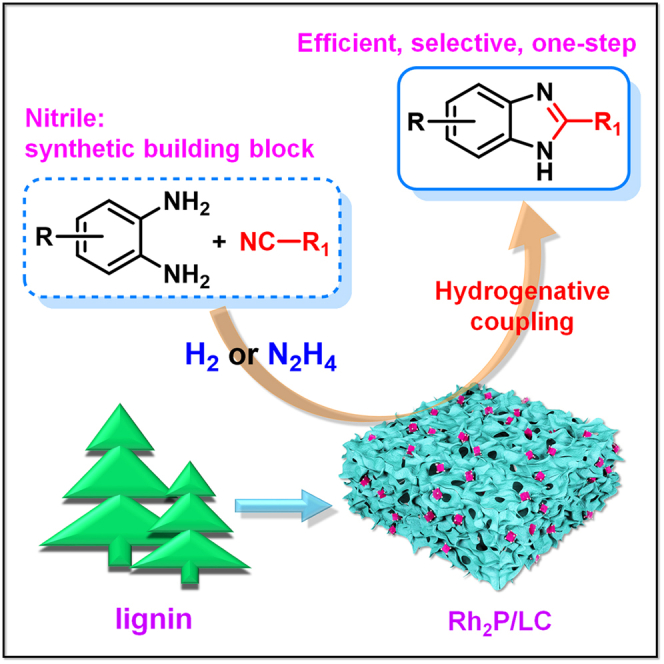

## Introduction

**Scheme 1:**
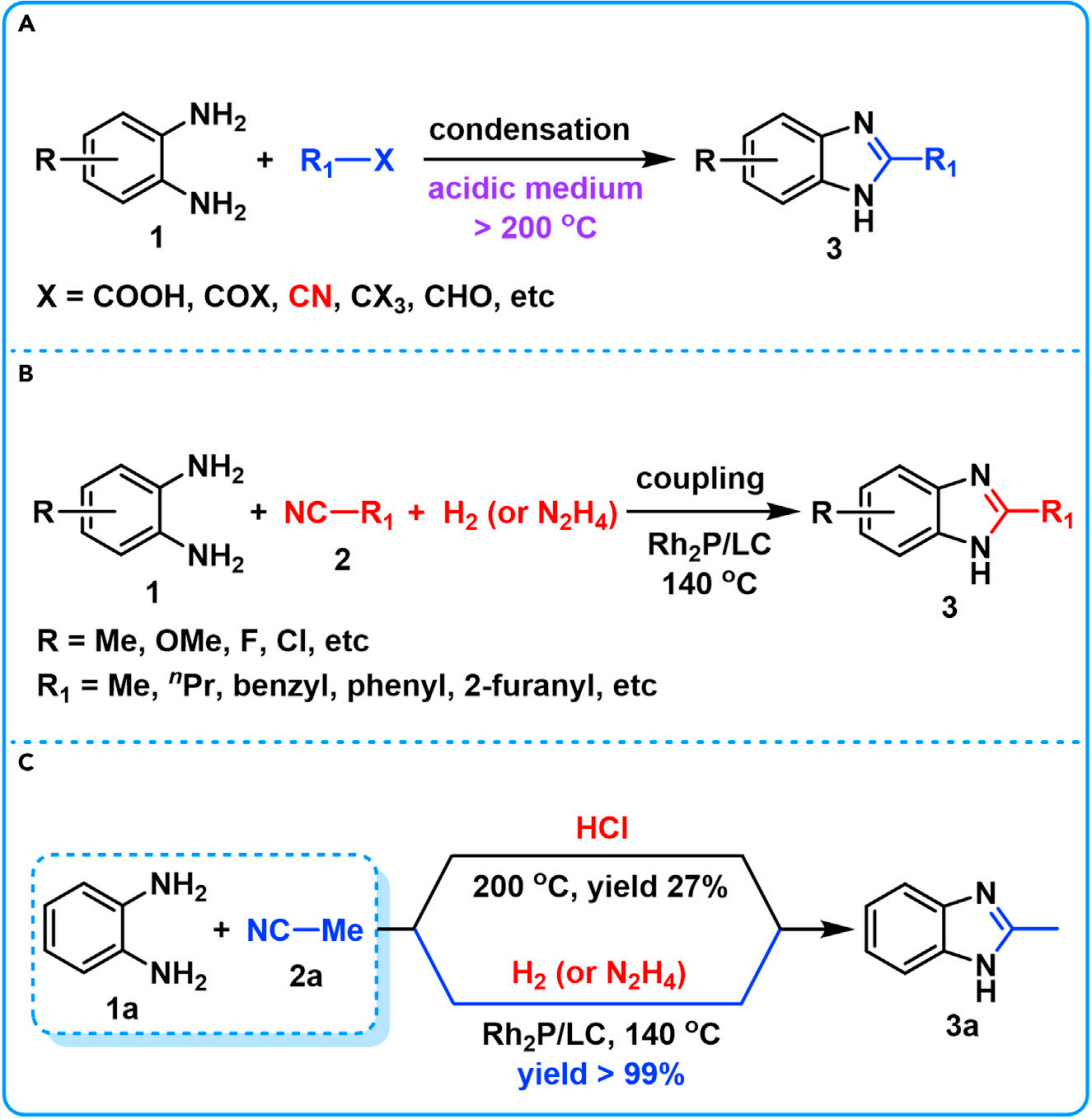
Methods for benzimidazole syntheses (A) Traditional condensation. (B) Present reductive coupling. (C) Comparison of condensation and coupling method.

Benzimidazole and its derivatives are pharmaceutically important heterocyclic compounds with a broad range of biological activities and pharmacological properties such as anti-viral, anti-fungal, anti-bacterial, anti-ulcer, anti-inflammatory, anti-hypertensive, anti-histaminic, anti-cancer, anti-tumor, and anti-HIV features (Chakrabarti et al., 2019; Keri et al., 2015). Their synthetic methods have received extensive attention due to their pharmaceutical importance. Benzimidazoles are traditionally prepared according to Ladenburg ring closure method (Scheme 1A) by direct condensation of 1,2-phenylenediamines (**1**) with carboxylic acids and their derivatives such as acids, acyl chlorides, anhydrides, aldehydes, amides and nitriles in the presence of strong acid at high reaction temperature. Among these carboxylic acid derivatives, due to a facile access to nitriles and their high availability as commodity chemicals, direct condensation of **1** with nitriles should have great potential in synthetic chemistry of benzimidazoles and in the productions of benzimidazole-related agrochemicals and pharmaceuticals (Dalziel et al., 2018; Le Questel et al., 2000; Tamura et al., 2013).

**Scheme 2:**
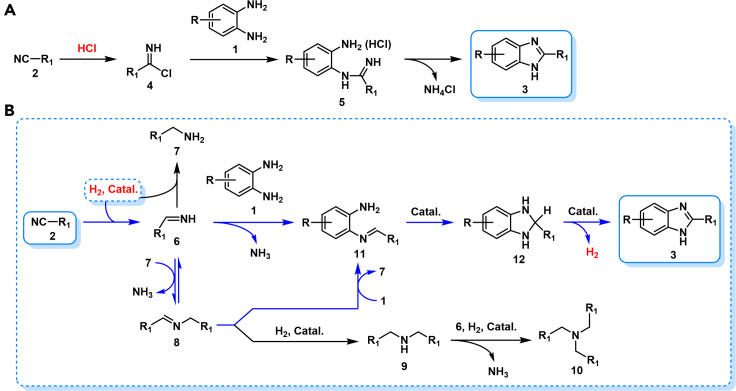
Proposed reaction mechanism for 3 formation (A) HCl-induced condensation. (B) transition-metal-promoted reductive coupling.

Unfortunately, research on direct condensation of **1** and nitriles is very limited. According to the reported results (Hölljes and Wagner, 1944), 27% yield of 2-methylbenzimidazole (**3a**) was obtained from 1,2-phenylenediamine (**1a**) and acetonitrile (**2a**) in the presence of an equivalent of anhydrous hydrogen chloride (HCl) in a sealed tube at 200°C for six hours (Scheme 1C). The corresponding condensation reaction mechanism suggests an initial formation of highly reactive ammonoacyl chloride (**4**, Scheme 2A) from nitrile (**2**) and HCl under anhydrous conditions. The *in situ* formed **4** smoothly promotes subsequent ring closure to yield benzimidazoles (**3**). Therefore, nitrile activation, *via* additively uniting with HCl (**4**, Scheme 2A) at 200°C, is a prerequisite for the condensation reaction. However, nitrile (C≡N bond) activation has been less explored so far when compared with C=C, C=O, C=N and O−N=O bonds owing to the high redox potential of nitriles and the low dissociation energy of C−CN bond.

Most recently, nitrile hydrogenation was developed for atom-economic synthesis of amine with transition-metal-based catalysts (Bagal and Bhanage, 2015; Werkmeister et al., 2014; Liu et al., 2018a; Nandi et al., 2017) (Table S1). However, a crucial selectivity issue arises from an inevitable formation of mixtures of primary (**7**), secondary (**9**) and tertiary amines (**10**) with alkylimines (**6**) and dialkylimines (**8**) as the proposed reactive intermediates (Scheme 2B). High reactivity of these reaction intermediates (**6** and **8**) competitively induces a series of parallel and consecutive reactions, resulting in a challenge of selectivity control and product separation. Generally, nitrile hydrogenation can be performed under relatively mild reaction conditions (20–140°C). We thus think that the presence of **1** in the nitrile-hydrogenation system should be able to trap the *in situ* formed two reactive imines intermediates (**6** and **8**) to give *N*-(*o*-aminopheny)-imine intermediate (**11**, Scheme 2B). A subsequent cyclization of **11** and successive dehydrogenation of the resulting ring-closing product (**12**, Scheme 2B) should yield **3** under the reaction conditions (Scheme 2B). Therefore, a transition-metal-promoted reductive coupling of nitriles and 1,2-phenylenediamines was investigated in this research for green and atom-economic synthesis of benzimidazoles (Scheme 2B).

As shown in Scheme 2B, the reductive coupling process should be initialized from catalytic hydrogenation of nitriles. Both heterogeneous and homogeneous catalysts were reported for nitriles hydrogenation. Precious metal-complex-based homogeneous catalysts evidently show excellent catalytic performance on the hydrogenation (Bagal and Bhanage, 2015; Werkmeister et al., 2014; Chakraborty and Berke, 2014; Hou et al., 2020; Islam et al., 2010; Liu et al., 2018b; Chakraborty and Milstein, 2017). Although the developed heterogeneous catalysts are very limited and generally suffer from low activity, low selectivity, and low tolerance to functional group when compared with homogeneous catalysts (Wang et al., 2019; Huang and Sachtler, 1999a, 1999b; Zhang et al., 2019; Monguchi et al., 2017; Li et al., 2012; Carothers and Jones, 1925; Braos-García et al., 2010). Efforts to explore efficient catalytic systems with excellent recyclability have always been going on for industrial purpose.

Rhodium-phosphine complexes are highly efficient for nitrile hydrogenation among various investigated homogeneous catalysts. While, rhodium phosphide (Rh_2_P) crystal shows surface Rh atoms surrounded by two coordinated P atoms, which is sterically and structurally similar to the Rh-P interactions in bisphosphine ligand-modified Rh complexes. Moreover, integrating P atoms into the lattices of Rh metal can tune its internal electronic structure, thus improving the intrinsic catalytic activity of the resulting Rh_2_P catalyst (Shi and Zhang, 2016; Zhuang et al., 2016). Currently, Rh_2_P is developed as an excellent heterogeneous catalyst for hydrogenation, hydrodeoxygenation, hydroformylation, hydrodesulfurization, and hydrodenitrogenation (Luo et al., 2020; Griffin et al., 2017; Alvarado Rupflin et al., 2017; Hayes et al., 2010).

**Figure 1:**
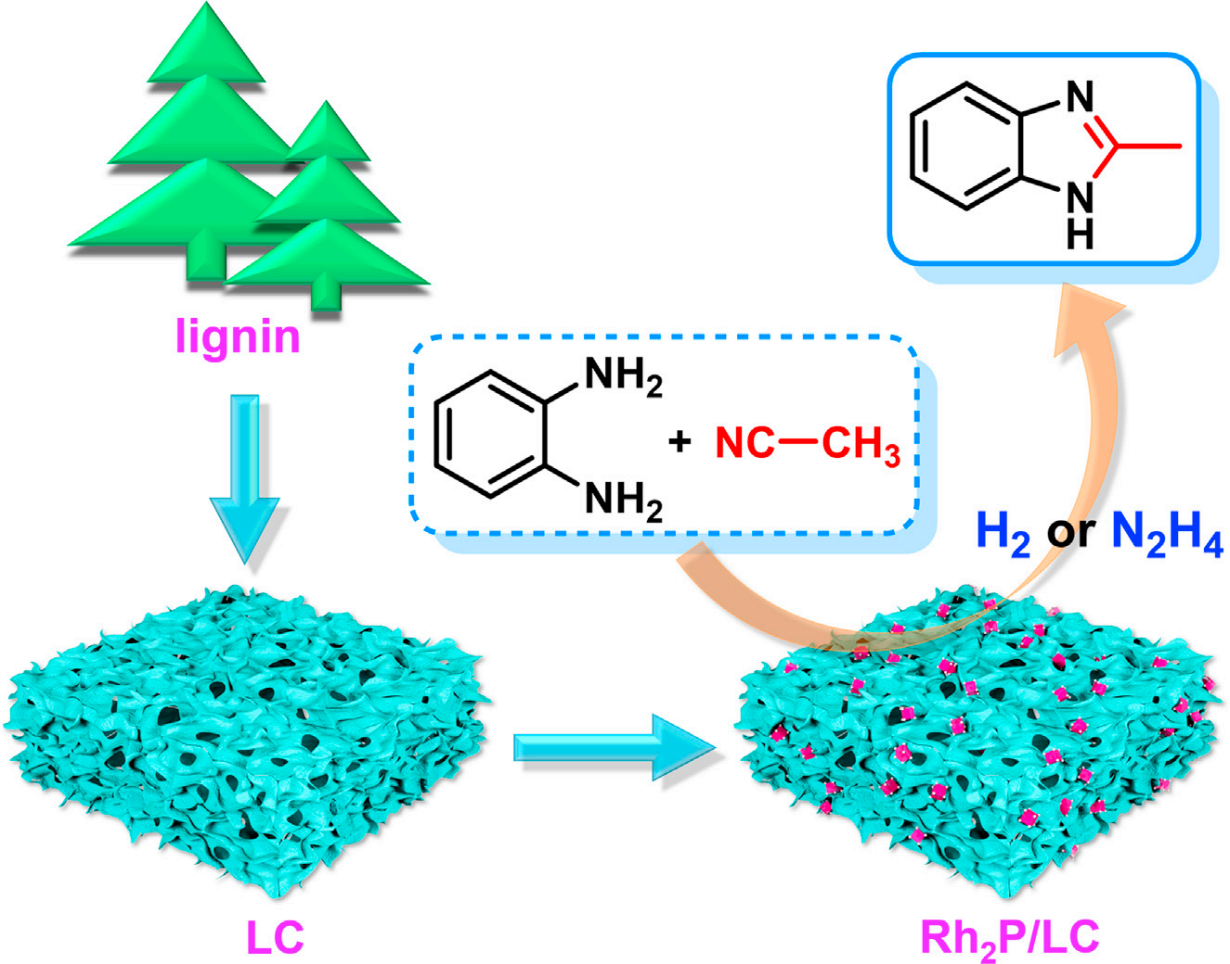
Rh_2_P/LC-promoted reductive coupling 1a with 2a

Herein, we demonstrated the first example of a reductive coupling of nitriles and 1,2-phenylenediamines to 2-alkylbenzimidazoles (Scheme 1B) by using Rh_2_P catalyst (Rh_2_P/LC) supported on lignin-derived hierarchically porous carbon (LC, Figure 1). The lignin was selected as precursor of the catalyst support due to its unique structure of three-dimensional and porous framework, rich in carbon-oxygen-based functional groups on the framework, scalable and renewable nature as a carbon-based feedstock (Zhu et al., 2019; Chatterjee and Saito, 2015). The developed Rh_2_P/LC demonstrates high efficiency by coupling serial tandem reactions of nitrile hydrogenation, **11** cyclization and **12** dehydration in one-pot (Scheme 2B). In contrast to traditional condensation method (Feng et al., 2016; Wade et al., 2015; Shiraishi et al., 2010; Wang et al., 1997; Selvam et al., 2009) (Table S2), Rh_2_P/LC promoted reductive coupling of **1a** and **2a** can readily perform at 140°C with >99% yield of **3a** by using H_2_ or hydrazine hydrate (N_2_H_4_⋅H_2_O) as hydrogen sources (Scheme 1C).

## Results and discussion

### Catalyst preparation and characterization

In this research, LC was used as catalyst support, which was obtained by calcination a mixture of enzymatic hydrolysis lignin (EHL) and potassium bicarbonate (KHCO_3_) at 800°C under atmospheric N_2_, followed by thoroughly leaching with aqueous HCl solution. While, Rh_2_P/LC_400_ catalyst was prepared by co-loading RhCl_3_ and bis(diphenylphosphino)ethane (dppe) ligand on the resulting LC surface, followed by a pyrolysis at 400°C under atmospheric H_2_/N_2_. The subscript 400 in Rh_2_P/LC_400_ indicates the final calcination temperature for catalyst preparation. The introduced dppe ligand provides P source for the formation of Rh_2_P species on the LC support during calcination procedure. For comparison, Rh catalyst (Rh/LC_400_) supported on LC was prepared without addition of the dppe ligand to investigate the electronic effect of the introduced P on the catalytic performance of the resulting Rh_2_P catalyst. Moreover, to understand the influence of metallic site on the reductive coupling, Pd (Pd/LC_400_) and Ru (Ru/LC_400_) catalysts were synthesized with same method to Rh_2_P/LC_400_; however, the expected metal phosphides of Pd-P and Ru-P samples were undetected on the resulting catalyst surfaces.

**Figure 2:**
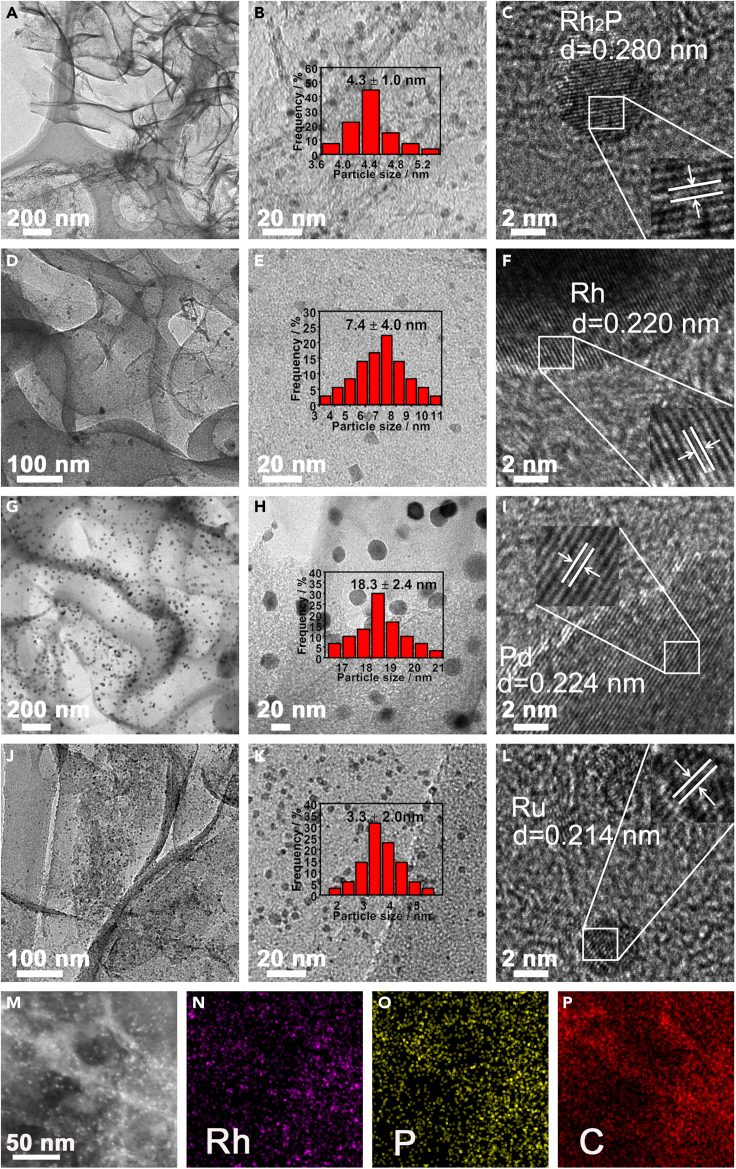
TEM and TEM-EDX characterizations (A–C) TEM of Rh_2_P/LC_400_. (D–F) TEM of Rh/LC_400_. (G–I) TEM of Pd/LC_400_. (J–L) TEM of Ru/LC_400_. (M–P) TEM-EDX mapping of Rh_2_P/LC_400_. Inserts of (B), (E), (H), (K) show the size distribution histogram by statistical analysis of 200 corresponding nanoparticles.

The morphology of the obtained samples was initially characterized by scanning electron microscopy (SEM) and transmission electron microscopy (TEM). EHL shows a bulky, solid, and compact architecture with irregular shapes and rough surface based on the SEM analysis (Figure S1A). While, the resulting LC exhibits a sponge-like structure with readily accessible, highly crosslinked and randomly opened macropores (Figures S1B and S1C). After Rh_2_P loading, the obtained Rh_2_P/LC_400_ possesses similar SEM micrograph to that of LC (Figure S1D). The TEM of Rh_2_P/LC_400_ exhibits ultrathin carbon nanosheet-assembled three-dimensional (3D) network with a crumpled, wrinkled, and rippled structure (Figure 2A). Additionally, Rh, P, and C elements are homogeneously and highly dispersed on the detected area of LC surface as shown by the TEM energy-dispersive X-ray (EDX) images of Rh_2_P/LC_400_ (Figure 2M–2P). The estimated average nanoparticle size of Rh_2_P was 4.3 nm (Figure 2B) with a detectable crystal fringe spacing of 0.280 nm, corresponding to the (200) crystal plane of Rh_2_P (Su et al., 2020) (Figure 2C).

In the case of Rh/LC_400_, its TEM images reveal a mean Rh nanoparticle size of 7.4 nm (Figures 2D and 2E) with lattice fringe spacing of 0.220 nm (Figure 2F), which can be indexed to the (111) plane of the Rh (Su et al., 2020). The TEM images of Pd/LC_400_ indicate a significantly increased average size to 18.3 nm for Pd nanoparticle on the LC support (Figures 2G and 2H). The observed lattice fringe spacing was detected as 0.224 nm, belonging to the (111) plane of Pd (Sun et al., 2020) (Figure 2I). Finally, for Ru/LC_400_ sample, TEM images show an average nanoparticle size of 3.3 nm (Figures 2J and 2K) with the lattice fringe spacing around 0.214 nm, corresponding to the (002) plane of Ru (Wang et al., 2020) (Figure 2L). Therefore, the average nanoparticle size decreased in the order of Pd > Rh > Rh_2_P > Ru among the investigated samples. Moreover, Rh_2_P/LC_400_ exhibits much smaller size of Rh nanoparticle with more homogeneous and more uniform dispersion if compared with Rh/LC_400_ (Figures 2A–2F). The observed porous architecture of Rh_2_P/LC_400_ should be favorable for mass transfer and diffusion in the investigated hydrogenative coupling reaction.

**Figure 3:**
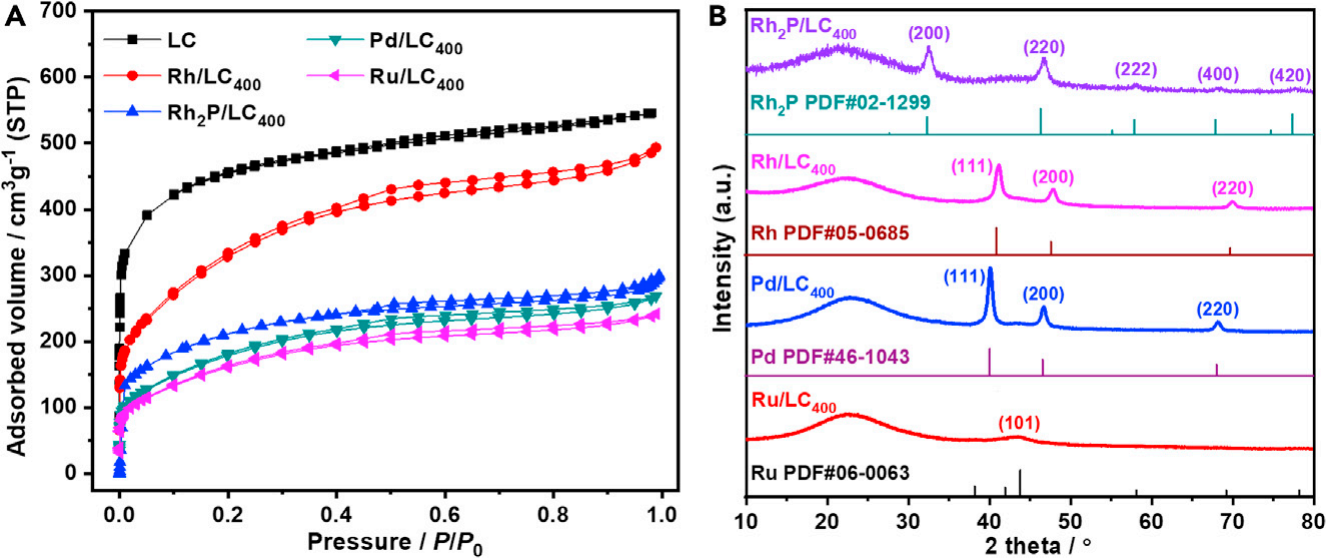
N_2_ sorption and XRD characterizations (A) N_2_ adsorption-desorption isotherm. (B) XRD patterns.

The textural properties of the developed samples were investigated with N_2_ sorption isotherm (Figure 3A), Table 1 lists the resulting results. LC support exhibits a steep rise at low *P*/*P*_0_ zone (*P*/*P*_0_ < 0.1) with a very weak hysteresis loop from middle to high *P*/*P*_0_ zone (Figure 3A). Therefore, LC has a micropore-prevailing and hierarchically micro-mesoporous morphology (Deng et al., 2015) (Figure S2) showing a specific Brunauer-Emmet-Teller (BET) surface area around 1,664 m^2^ g^−1^. After loading with transition metal, the resulting metallic catalyst exhibits a significantly reduced specific surface area (Table 1). For example, Rh_2_P/LC_400_ exhibits a specific surface area of 724 m^2^ g^−1^ (Table 1).

**Table 1:** Textural parameters of the investigated samples

Sample	*S*_BET_^a^ [m^2^ g^−1^]	*S*_micro_^b^ [m^2^ g^−1^]	*S*_meso_^c^ [m^2^ g^−1^]	*D*_micro_/*D*_meso_^d^ [nm]	*V*_total_^e^ [cm^3^ g^−1^]	*V*_micro_/*V*_meso_^f^ [cm^3^ g^−1^]
LC	1664	1320	344	0.78/2.27	1.20	0.55/0.65
Rh_2_P/LC_400_	724	176	548	1.02/4.02	0.45	0.08/0.37
Rh_2_P/LC_600_	505	178	327	1.03/4.01	0.62	0.08/0.54
Rh_2_P/LC_800_	696	232	464	1.01/4.00	0.71	0.11/0.60
Rh/LC_400_	1156	484	672	0.85/3.11	0.69	0.21/0.48
Pd/LC_400_	630	135	495	0.85/3.08	0.41	0.12/0.29
Ru/LC_400_	567	274	293	0.85/3.10	0.37	0.10/0.27
recovered Rh_2_P/LC_400_	426	195	231	0.60/2.05	0.33	0.10/0.23
recovered Rh/LC_400_	797	325	472	1.01/3.99	0.57	0.16/0.41

a *S*_BET_, specific surface area.

b *S*_micro_, the specific surface area of micropore.

c Smeso, the specific surface area of mesopore.

d Dmicro/Dmeso, the average diameters of micropore (Dmicro) and mesopore (Dmeso).

e Vtotal, the total specific pore volume.

f Vmicro/Vmeso, the specific pore volume of micropore (Vmicro) and mesopore (Vmeso).

All of the obtained samples were then performed with X-ray diffraction (XRD) analysis. Rh_2_P/LC_400_ shows a broad diffraction peak at 2*θ* = 21.0° (Figure 3B), which is indexed to the diffraction peak from amorphous carbon. In addition, five characteristic peaks at 2*θ* = 32.5, 46.8, 58.0, 68.2, and 77.8° are respectively assigned to the (200), (220), (222), (400), and (420) planes of Rh_2_P (JCPDF file no. 02-1299), suggesting the presence of Rh_2_P crystal on the LC surface (Duan et al., 2017). In the case of Rh/LC_400_, three representative diffraction peaks at 2*θ* values of 41.1, 47.8 and 69.9° (Figure 3B) are individually indexed to the (111), (200), and (220) planes of metallic Rh (JCPDF file no. 05-0685) (Kundu et al., 2018), which indicates successful loading of metallic Rh on the LC surface.

Pd/LC_400_ displays three characteristic peaks at 2*θ* = 40.2, 46.7, and 68.2° (Figure 3B), which are respectively assigned to the (111), (200), and (220) planes of metallic Pd (JCPDF file no. 46-1043). Therefore, Pd(0) crystals, rather than palladium phosphide, are suggested to be deposited on the LC surface for the obtained Pd/LC_400_ (Li et al., 2016). Notably, Ru/LC_400_ only displays a weak characteristic peak at 2*θ* = 44.2° (Figure 3B), corresponding to the crystalline phases of Ru (JCPDF file no. 06-0063). This observation can presumably be attributed to the highly dispersed and homogeneous Ru species with small particle size on the LC surface (Zou et al., 2019).

**Figure 4:**
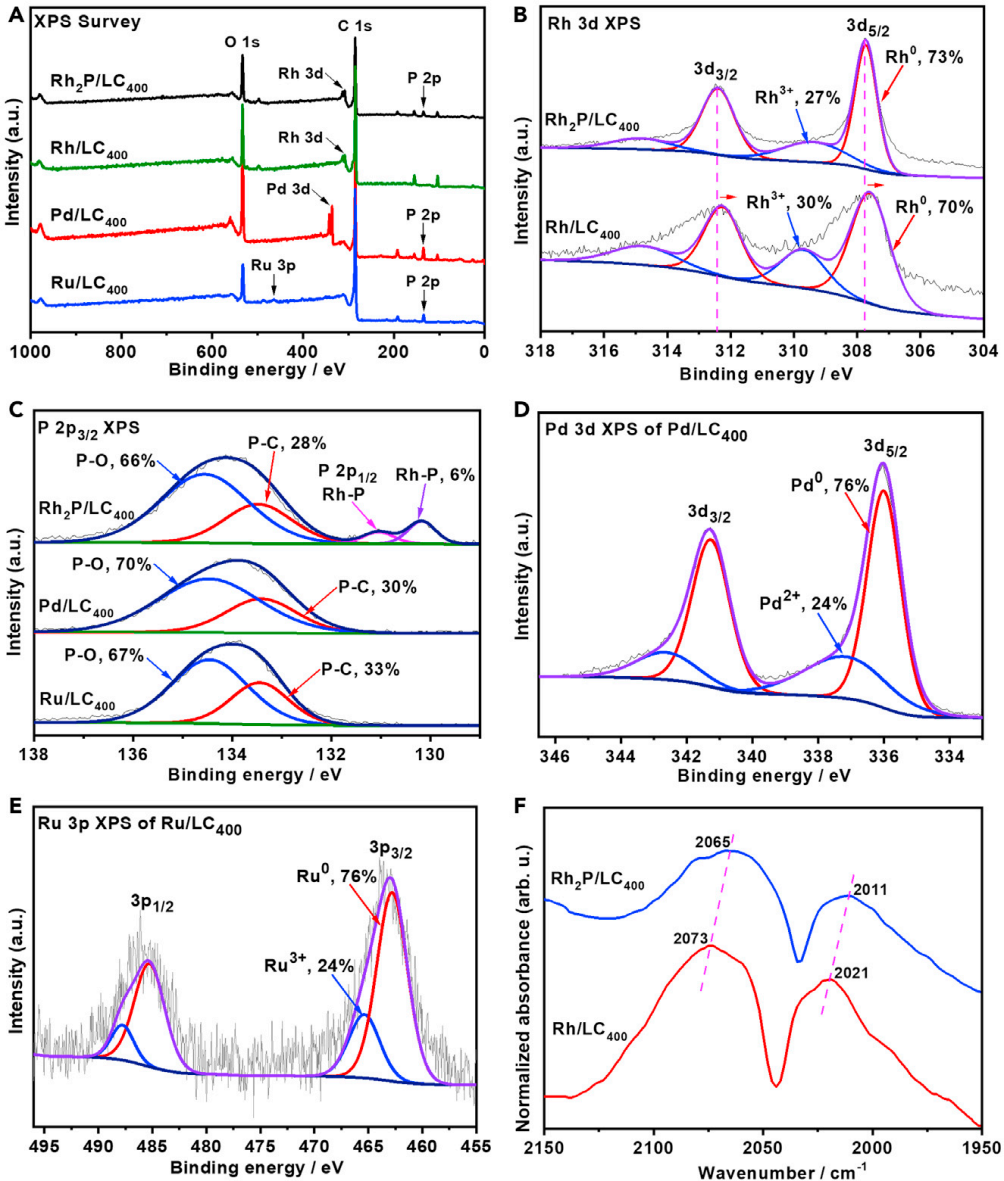
XPS and CO-probed FT-IR characterizations (A) XPS scan survey of Rh_2_P/LC_400_, Rh/LC_400_, Pd/LC_400_ and Ru/LC_400_. (B) Rh 3d XPS of Rh_2_P/LC_400_ and Rh/LC_400_. (C) P 2p_3/2_ XPS of Rh_2_P/LC_400_, Pd/LC_400_ and Ru/LC_400_. (D) Pd 3d XPS of Pd/LC_400_. (E) Ru 3p XPS of Ru/LC_400_. (F) CO-probed FT-IR spectra for Rh_2_P/LC_400_ and Rh/LC_400_.

Surface elements and their chemical states of the obtained samples were further examined by X-ray photoelectron spectroscopy (XPS). For Rh_2_P/LC_400_, the presence of Rh, P, C, and O elements is confirmed in the XPS survey (Figure 4A and Tables S3–S7). The high-resolution Rh 3d XPS of Rh_2_P/LC_400_ can be generally deconvolved into two sets of doublet peaks (Figure 4B). The set of strong doublet peaks, located at binding energies of 312.4 eV (indexed to Rh 3d_3/2_) and 307.7 eV (indexed to Rh 3d_5/2_), can be ascribed to metallic Rh, corresponding to Rh_2_P species (Luo et al., 2020). The other set of weak doublet peaks at 314.8 eV (indexed to Rh 3d_3/2_) and 309.8 eV (indexed to Rh 3d_5/2_) are ascribed to the Rh(III) oxidation state (Luo et al., 2020). Rh_2_P (73%) is formed as the predominant species on the Rh_2_P/LC_400_ surface based on the integration areas of these two doublets, accordingly demonstrating successful formation of Rh_2_P catalyst from Rh-dppe complex under the synthetic conditions. High-resolution P 2p_3/2_ XPS of Rh_2_P/LC_400_ (Figure 4C) can be deconvoluted into three characteristic peaks at the binding energy of 134.5 eV (indexed to P−O), 133.3 eV (indexed to P−C), and 130.1 eV (indexed to Rh−P). The presence of P−O species presumably originates from oxidation of the surface P upon exposure to air, whereas the existence of P−C indicates the formation of doped P atom into the LC matrix (Chen et al., 2016). Finally, the formation of Rh_2_P species can be demonstrated by the presence of Rh−P species from the P 2p_3/2_ XPS of Rh_2_P/LC_400_. Therefore, our XPS analysis of Rh_2_P/LC_400_ is in accordance with its TEM and XRD results, confirming the major Rh_2_P species on the Rh_2_P/LC_400_ surface.

In the case of Rh/LC_400_, its high-resolution Rh 3d XPS can be deconvolved into two sets of doublet peaks (Figure 4B). The set of strong doublet peaks, located at binding energies of 312.3 eV (indexed to Rh 3d_3/2_) and 307.6 (indexed to Rh 3d_5/2_), can be ascribed to metallic Rh as a prevailing species (70%), whereas the set of weak doublet peaks at 314.7 eV (indexed to Rh 3d_3/2_) and 309.7eV (indexed to Rh 3d_5/2_) are ascribed to the Rh(III) oxides. Notably, Rh_2_P/LC_400_ shows evident shifts to higher binding energies in the Rh 3d XPS if compared with those of Rh/LC_400_ (Figure 4B). The observed positive shifts in the Rh 3d XPS indicate enhanced charge transfer to Rh as well as stronger interactions between P and Rh in the Rh_2_P/LC_400_ sample (Su et al., 2020). The Rh-P interaction in Rh_2_P can modify the surface charge states and electron cloud density on the Rh site, which should be beneficial to H_2_ activation.

For Pd/LC_400_ sample, its high-resolution Pd 3d XPS are deconvolved into two sets of doublet peaks (Figure 4D). The doublet peaks with strong intensity, located at a binding energy of 341.2 eV (indexed to Pd 3d_3/2_) and 336.0 eV (indexed to Pd 3d_5/2_), can be assigned to metallic Pd as a prevailing species (76%). The set of weak doublet peaks at 342.2 eV (indexed to Pd 3d_3/2_) and 337.0 eV (indexed to Pd 3d_5/2_) are ascribed to the Pd(II) oxides (Li et al., 2016). The high-resolution P 2p_3/2_ XPS of Pd/LC_400_ (Figure 4C) can be deconvoluted into two peaks, which are ascribed to P−O (134.5 eV) and P−C (133.3 eV) species.

In the case of Ru/LC_400_, the most intensive photoemission line of Ru 3d is strongly overlapped with C 1s line from the LC support. The surface Ru species were thus examined with Ru 3p XPS. The high-resolution Ru 3p XPS were fit into two sets of doublet peaks (Figure 4E). The strong doublet peaks with binding energy of 484.0 eV (indexed to Ru 3p_1/2_) and 461.5 eV (indexed to Ru 3p_3/2_), can be assigned to metallic Ru as a predominant species (76%) (Li et al., 2020a). The set of weak doublet peaks at 486.5 eV (indexed to Ru 3p_1/2_) and 464.0 eV (indexed to Ru 3p_3/2_) are ascribed to Ru(III) oxides (Zhao et al., 2020). The high-resolution P 2p_3/2_ XPS peaks of Ru/LC_400_ is very close to that of Pd/LC_400_ as described above (Figure 4C).

Notably, palladium phosphide and ruthenium phosphide species were unobserved on the surfaces of Pd/LC_400_ and Ru/LC_400_, respectively, based on TEM, XRD, and XPS analysis. Therefore, the XPS analysis further suggests that only Rh_2_P/LC_400_ sample contains Rh_2_P species on the LC surface although the synthetic procedure is the same for Rh_2_P/LC_400_, Pd/LC_400_ and Ru/LC_400_ samples. In the cases of Pd/LC_400_ and Ru/LC_400_ samples, the expected metal phosphide species are unobserved on the LC surface.

### Hydrogenative coupling reaction

**Figure 5:**
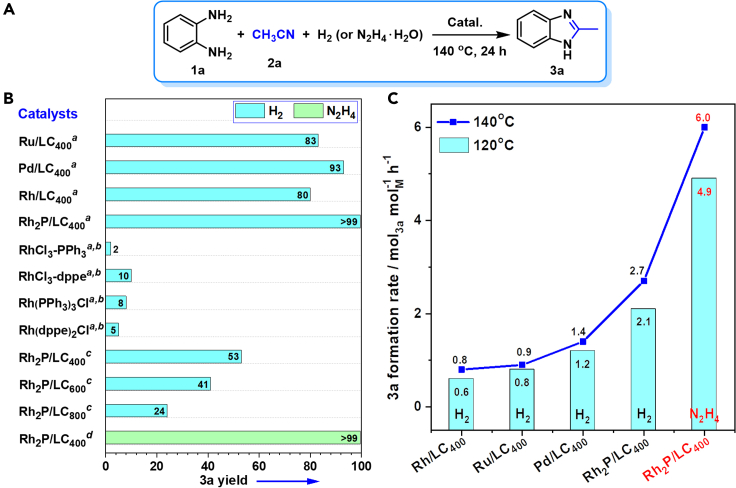
Catalyst screen for hydrogenative coupling (A) Hydrogenative coupling of **1a** with CH_3_CN to **3a**. (B) Comparison of catalyst activity based on one run of each catalyst. (C) **3a** formation rate over various catalysts. ^*a*^Performed with catalyst (20 mg), **1a** (0.3 mmol), *P*_H2_ (1.0 MPa), CH_3_CN (3.0 mL); ^*b*^based on an equimolar amount of the Rh site in the Rh_2_P/LC_400_ [Rh (8.0 × 10^−3^ mmol) and P (3.2 × 10^−2^ mmol)]; ^*c*^catalyst (10 mg); ^*d*^N_2_H_4_⋅H_2_O (1.0 mmol), *P*_N2_ (1.0 MPa), *t* (6 h).

The hydrogenative coupling of 1,2-phenylenediamine (**1a**) and acetonitrile (**2a**) for 2-methyl-1*H*-benzo[*d*]imidazole (**3a**) synthesis with H_2_ as hydrogen source was investigated as a model reaction to optimize reaction conditions with the obtained catalysts (Figures 5A and 5B). **3a** was unobserved under catalyst free conditions, indicating the absence of catalytic active sites for the coupling reaction. Rh/LC_400_ gave 80% yield of **3a**, which is very close to the catalytic activity of Ru/LC_400_ (83% yield, Figure 5B). A significantly increased **3a** yield to 93% was obtained by Pd/LC_400_. However, among the investigated catalysts, Rh_2_P/LC_400_ was the most efficient one by producing quantitative yield (>99%) of **3a**.

The Rh_2_P/LC_400_ was obtained by pyrolysis of a mixture of RhCl_3_, dppe and LC under H_2_/N_2_ at 400°C. Therefore, to probe the catalytic site of Rh_2_P/LC_400_, a mixture of RhCl_3_−PPh_3_ (PPh_3_, triphenyl phosphine) and RhCl_3_−dppe were respectively investigated as homogeneous catalysts for the coupling (Figure 5B). However, negligible **3a** yields (2–10%) were obtained with the above homogeneous system. Moreover, the reductive coupling was further carried out with Rh-based classical hydrogenation catalysts such as Rh(PPh_3_)_3_Cl and Rh(dppe)_2_Cl, which, again, yielded very limited amount of **3a** (5–8% yield, Figure 5B). It is thus believed that Rh_2_P species, instead of Rh-P based complexes, is the true active site on the Rh_2_P/LC_400_ surface for the reductive coupling reaction.

To understand the influence of pyrolysis temperature on the catalytic performance of the catalyst, the reductive coupling reaction were performed with a low loading level of Rh_2_P/LC catalysts obtained at 400°C-pyrolysis (denoted as Rh_2_P/LC_400_), 600°C-pyrolysis (denoted as Rh_2_P/LC_600_), and 800°C-pyrolysis (denoted as Rh_2_P/LC_800_). Evidently, Rh_2_P/LC_400_ is the most effective one for the reaction (Figure 5B). Characterization of Rh_2_P/LC_600_ and Rh_2_P/LC_800_ are almost the same with Rh_2_P/LC_400_ (Figures S1–S3). However, a significantly increased mean nanoparticle size of Rh_2_P was observed with 8.6 nm for Rh_2_P/LC_600_ and 12.6 nm for Rh_2_P/LC_800_, respectively, which may presumably lead to the reduced activity. Finally, in addition to H_2_, N_2_H_4_⋅H_2_O was an alternative excellent hydrogen source for the reductive coupling reaction in the presence of Rh_2_P/LC_400_ catalyst by producing quantitative yield of **3a** (>99%, Figure 5B).

The catalytic activities of various catalysts were further quantitatively compared based on formation rate of **3a**. In this research, **3a** formation rate in the hydrogenative coupling was obtained under a low **3a** yield around 10–15%, given as the amount of formed **3a** per amount of metal sites per hour for the investigated catalyst. Figure 5C demonstrates a comparable activity between Rh/LC_400_, Ru/LC_400_, and Pd/LC_400_. Although Rh_2_P/LC_400_ was proved to be the most active catalyst among various investigated samples, increase of reaction temperature slightly enhanced **3a** formation rate. Moreover, Rh_2_P/LC_400_−N_2_H_4_⋅H_2_O system shows a doubled activity when compared with the Rh_2_P/LC_400_−H_2_ system under investigated conditions. Therefore, Rh_2_P/LC_400_ exhibits superior catalytic activity over Rh/LC_400_, presumably due to the electronic effect of Rh on the hydrogenative coupling.

**2a** thus functions as both reagent and solvent with an excess amount for the hydrogenative coupling under the above reaction conditions. In fact, the hydrogenative coupling can be well performed in tetrahydrofuran (THF) solvent. The influence of **2a** concentration in THF on the coupling revealed that **3a** yields increased with **2a** concentration up to 92% with an optimal **2a** concentration of 1.0 mmol mL^−1^ in THF, corresponding to 10 molar equivalents of **2a** to **1a** (Figure S4). Notably a maximal **3a** yield of 96% was obtained in THF with the **2a**/**1a** molar ratio of 30. The effect of reaction solvent on the coupling reaction demonstrated that both **2a** and THF were effective for **3a** formation (Table S8).

### Electronic effect of Rh_2_P

The electronic state of Rh species on Rh_2_P/LC_400_ and Rh/LC_400_ was then investigated and compared by using Fourier transform infrared spectroscopy (FT-IR) with carbon monoxide (CO) as a probe molecule. The FT-IR spectra of CO-adsorbed Rh_2_P/LC_400_ revealed two different CO coordination modes (Figure 4F). The band at 2065 cm^−1^ is indexed to typically terminal monocarbonyl species of Rh−CO, corresponding to the terminal CO adsorbed on metallic Rh of the Rh_2_P, whereas the band at 2011 cm^−1^ is ascribed to the dicarbonyl species of Rh(CO)_2_ (Alvarado Rupflin et al., 2017; Yates et al., 1979). In contrast, the corresponding CO coordination modes over Rh/LC_400_ are observed at 2073 cm^−1^ for Rh−CO and at 2021 cm^−1^ for Rh(CO)_2_, respectively. Evidently, Rh_2_P/LC_400_ shows blue-shifts in the CO-probed FT-IR spectra if compared with Rh/LC_400_ (Figure 4F). The observed blue-shift indicates that Rh species on the Rh_2_P/LC_400_ has weaker electron-donating capability than in the case of the Rh/LC_400_, which suggests a decrease of electron density on the Rh site in the Rh_2_P/LC_400_ due to P doping (Komanoya et al., 2017; Nakajima et al., 2013). Moreover, P doping leads to a decreased adsorption intensity of Rh_2_P/LC_400_ in the CO-probed FT-IR spectra when compared with those of Rh/LC_400_. Our Rh 3d XPS analysis for Rh_2_P/LC_400_ indicates an enhanced charge transfer to Rh as well as strong interactions between P and Rh in the sample (Figure 4B). Generally, the results from CO-probed FT-IR are in line with our Rh 3d XPS analysis, suggesting a decreased electron density on Rh species in the Rh_2_P/LC_400_. Therefore, the electronic state of Rh_2_P/LC_400_ may presumably affect adsorption and activation of H_2_, leading to an efficient reductive coupling.

### Isotope trace investigation

**Scheme 3:**
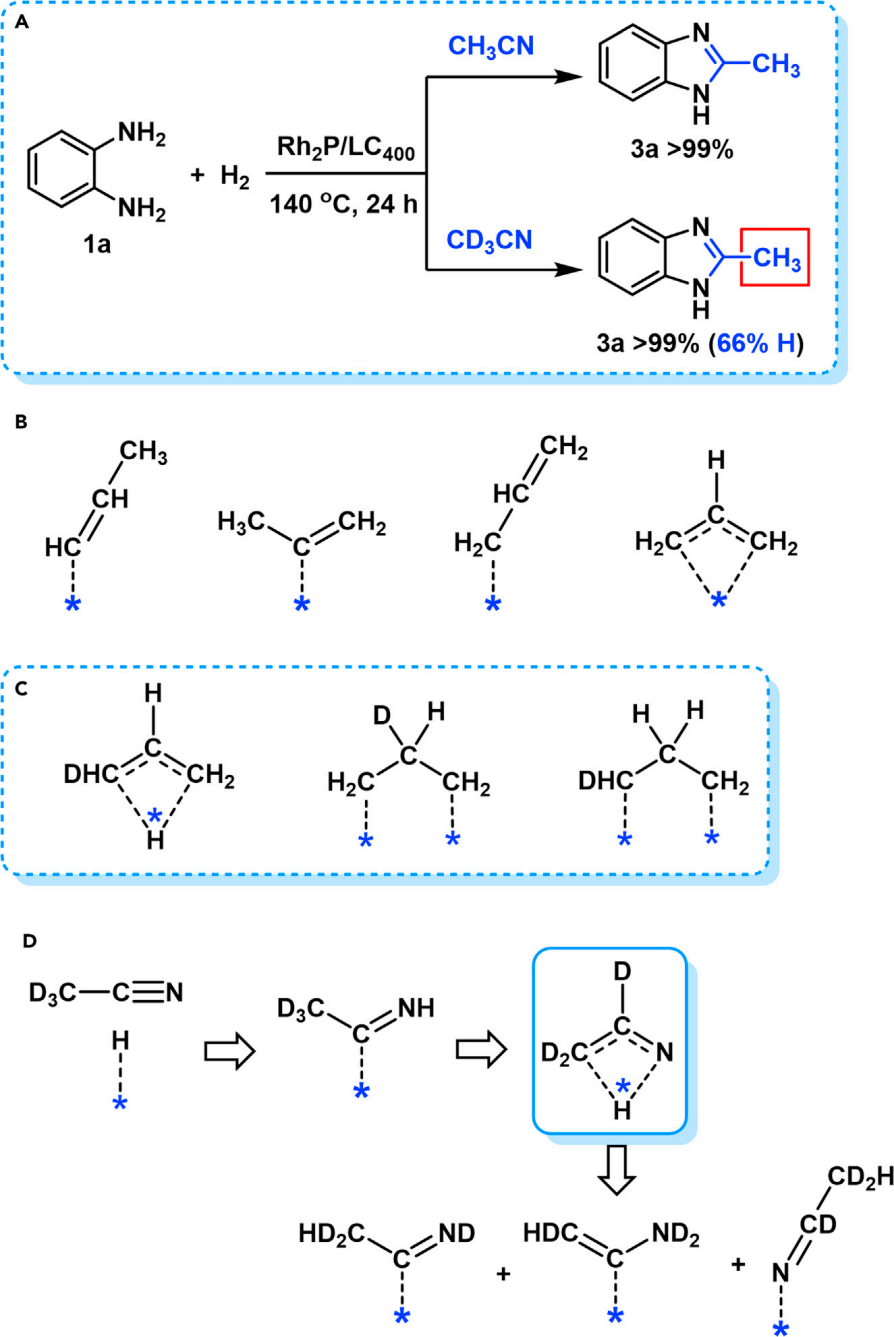
Isotope trace investigation (A) Hydrogenative coupling of 1a−CH_3_CN and 1a−CD_3_CN. Performed with 1a (0.3 mmol), Rh_2_P/LC_400_ (20 mg), *P*_H2_ (1.0 MPa), CH_3_CN, or CD_3_CN (3.0 mL). (B) Dissociative mechanism in C_3_H_6_–H_2_ reaction. (C) Intramolecular H-shift mechanism in C_3_H_6_–H_2_ reaction. (D) Proposed H/D exchange in CD_3_CN–H_2_ reaction.

**Figure 6:**
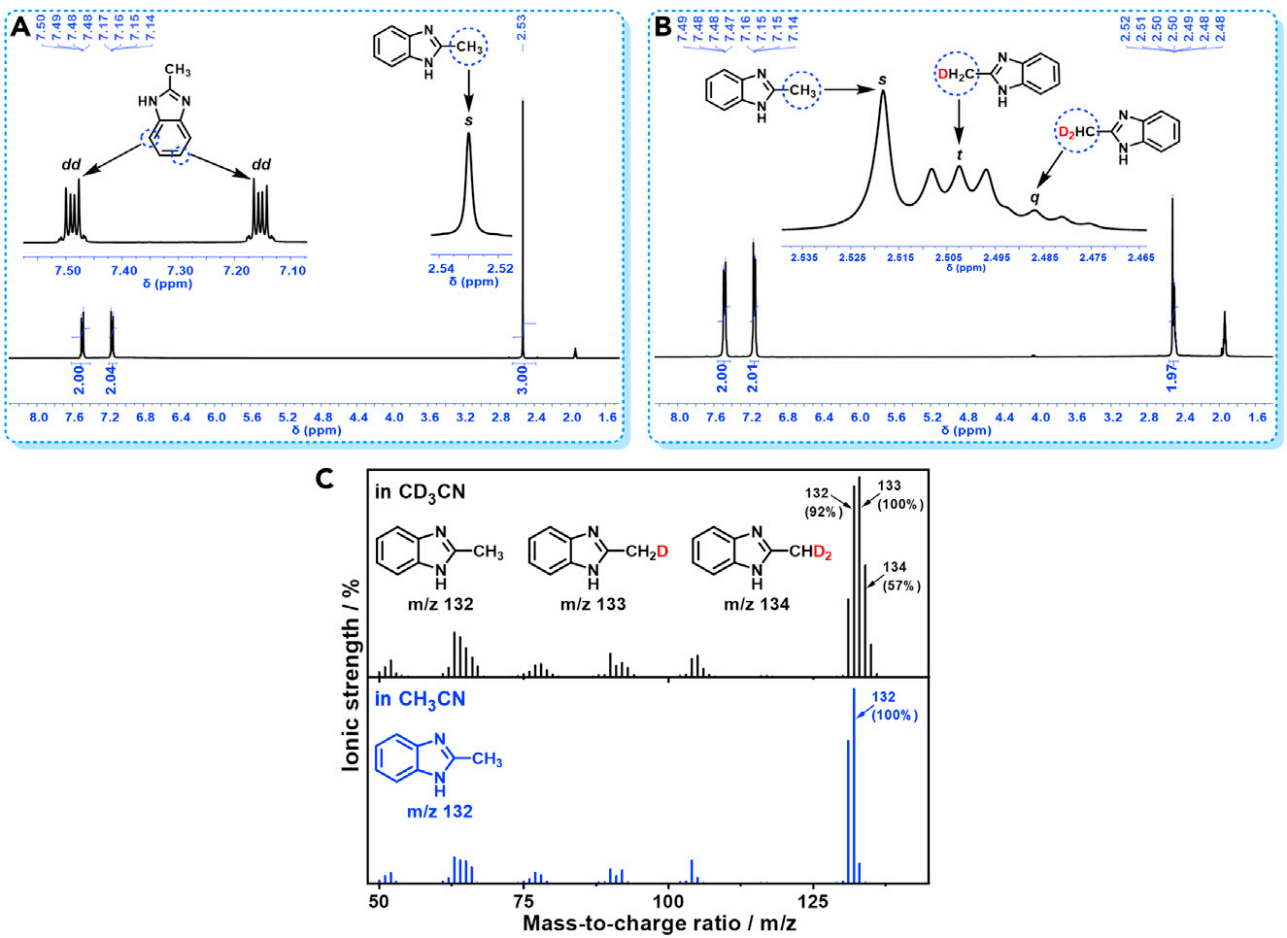
Comparison of 1a−CH_3_CN and 1a−CD_3_CN in hydrogenative coupling (A) ^1^H NMR spectra of **3a** from **1a**–CH_3_CN. (B) ^1^H NMR spectra of **3a** from **1a**–CD_3_CN. (C) MS of **3a** from **1a**–CH_3_CN and **1a**–CD_3_CN.

Hydrogenative couplings of **1a** with CH_3_CN and CD_3_CN were then respectively performed under H_2_ with Rh_2_P/LC_400_ catalyst. The proton nuclear magnetic resonance (^1^H NMR) and mass spectrometry (MS) were applied to monitor the H/D exchange in the coupling process (Scheme 3A). Figure 6A shows the resulting ^1^H NMR of the **3a** formed from **1a**−CH_3_CN, the peak of the –NH– from **3a** was unobserved. While the signals at 7.48 ppm (*dd*, *J*_HH_ = 3.2, 6.0 Hz, aromatic –CH–), 7.15 ppm (*dd*, *J*_HH_ = 3.2, 6.0 Hz, aromatic –CH–), 2.53 ppm (*s*, –CH_3_) were well separated in the ^1^H NMR of **3a**. The resulting relative integration intensity reflected a 2_(CH)_:2_(CH)_:3_(CH3)_ ratio of proton, thus confirming the chemical structure of 2-methyl-1*H*-benzo[*d*]imidazole for the **3a**. In the case of **3a** formed from **1a**−CD_3_CN, the observed aromatic –CH– signals were almost the same with those from **1a**−CH_3_CN; however, the resulting signals at methyl-group region were quite complicated (Figure 6B). In addition to a singlet at 2.52 ppm (–CH_3_), a 1:1:1 triplet (*J*_HD_ = 1.7 Hz) was observed at 2.50 ppm (–CH_2_D), and a 1:2:3:2:1 quintet (*J*_HD_ = 1.7, 3.4 Hz) was detected at 2.49 ppm (–CHD_2_) (Anet and O'Leary, 1989; Denny et al., 1994; Wheelhouse and Stevens, 1993; Horn and Everett, 1971). The above ^1^H NMR analysis indicates the formation of mono-protonated, di-protonated and tri-protonated methyl (−CD_n_H_3-n_) group in **3a** with a total of 66% proton incorporation at the inert methyl carbon atoms during the **1a**−CD_3_CN coupling. The molar ratio of –CH_3_, –CH_2_D and –CHD_2_ groups were 13:23:10 based on the corresponding integration areas (Figure 6B).

Figure 6C further compares the MS of **3a** formed from CH_3_CN and CD_3_CN. For the hydrogenative coupling of **1a**−CH_3_CN, the most abundant parent molecular ions for the resulting **3a** were observed at *m/z* = 132, suggesting formation of the expected 2-methyl-1*H*-benzo[*d*]imidazole. In contrast, the **3a**, formed in **1a**−CD_3_CN, contained at least three compounds of 2-methyl-1*H*-benzo[*d*]imidazole (*m/z* = 132), 2-(methyl-*d*_1_)-1*H*-benzo[*d*]imidazole (*m/z* = 133) and 2-(methyl-*d*_2_)-1*H*-benzo[*d*]imidazole (*m/z* = 134) with the –CH_2_D group-containing **3a** as the most predominant molecule. Our ^1^H NMR and MS analyses thus clearly indicate the appearance of proton at the methyl group (−CD_n_H_3-n_) of the formed **3a** *via* the hydrogenative coupling of **1a**−CD_3_CN, suggesting the presence of H/D exchange between the inert –CD_3_ group of CD_3_CN and H_2_ during the CD_3_CN-hydrogenation process.

Previously, propene–deuterium (C_3_H_6_–D_2_) reaction was extensively investigated with various transition metal catalysts and equally exchangeable of all the hydrogens in C_3_H_6_ were observed (Hirota and Hironaka, 1965; Naito and Tanimoto, 1999). The suggested reaction mechanism involved a rate-determining step of a D_2_ dissociation and subsequent incorporation of D atom into the dissociatively adsorbed intermediates such as *n*-propenyl, *sec*-propenyl and allylic species (Scheme 3B). Moreover, an intramolecular double-bond migration mechanism in propene between C1 and C3 carbon was suggested by a bridged hydrogen during the deuteration (Scheme 3C). In the case of reported CH_3_CN–D_2_ reaction, a series of deuterated amines such as (CD_3_CD_2_)_2_NH and (CH_3_CD_2_)NH(CD_2_CD_3_) were detected by an isotope exchange of D_2_ with the H atoms in –CH_3_ group of CH_3_CN (Huang and Sachtler, 1999a, 1999b). Therefore, in our case, an intramolecular D-shift may presumably promote H/D exchange between the –CD_3_ group of CD_3_CN and H_2_ during the hydrogenation (Scheme 3D).

### Controlled experiments and catalyst reusability

**Figure 7:**
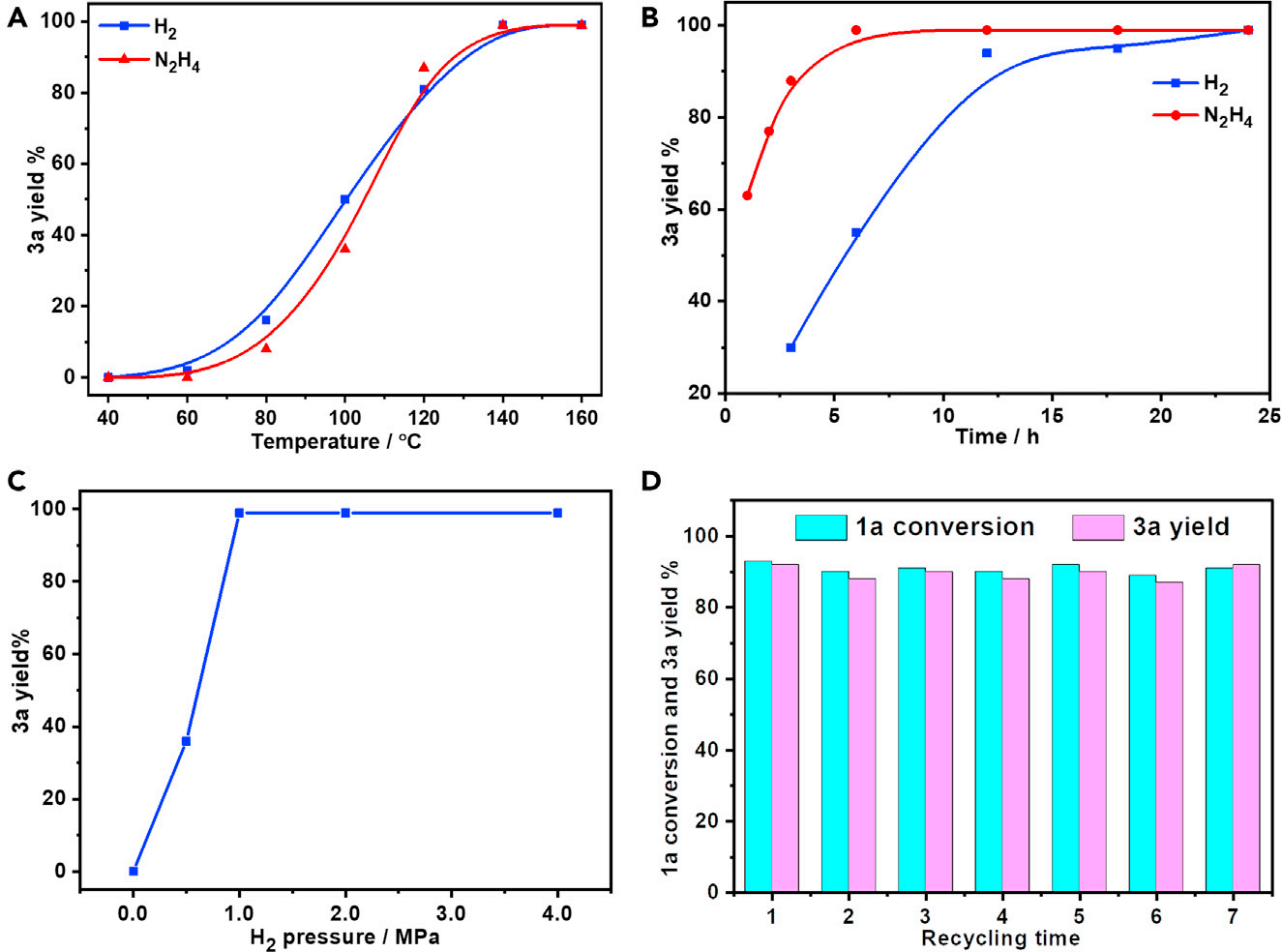
Reaction optimization and catalyst reusability (A) Reaction temperature. (B) Reaction time. (C) Initial H_2_ pressure. (D) Reusability of Rh_2_P/LC_400_. The recovered Rh_2_P/LC_400_ was collected by vacuum filtration, thoroughly washed with ethyl alcohol, dried under the vacuum, added into the autoclave, and performed with the previous reaction conditions. Performed with: (A) Rh_2_P/LC_400_ (20 mg), **1a** (0.3 mmol), CH_3_CN (3.0 mL), *P*_H2_ (1.0 MPa) or [*P*_N2_ (1.0 MPa) and N_2_H_4_⋅H_2_O (1.0 mmol)], *t* (24 h); (B) *T* (140 °C); (C) *t* (24 h), *T* (140°C); (D) Rh_2_P/LC_400_ (10 mg), **1a** (0.2 mmol).

Rh_2_P/LC_400_ catalyst was then selected for reductive coupling with both H_2_ and N_2_H_4_⋅H_2_O as hydrogen sources to probe the influence of reaction temperature and reaction time on the reaction. Reaction temperature significantly promotes the coupling reaction (Figure 7A). Negligible **3a** yields were observed below 80°C. While **3a** yields steeply increased with reaction temperature above 80°C with quantitative **3a** yield (>99%) obtained at 140°C. Regarding the influence of reaction time on the coupling (Figure 7B), N_2_H_4_⋅H_2_O can remarkably reduce reaction time to 6 h by giving >99% yield of **3a**. However, a significantly prolonged reaction time to 24 h was observed for H_2_ to yield quantitative **3a**. Finally, the effect of H_2_ pressure on the reaction revealed that the coupling cannot occur in the absence of H_2_, **3a** yield steeply increased with H_2_ pressure with an optimal H_2_ pressure of 1.0 MPa (Figure 7C).

The recyclability of Rh_2_P/LC_400_ was subsequently examined with the hydrogenative coupling for **3a** synthesis. The recovered Rh_2_P/LC_400_ was collected by vacuum filtration, thoroughly washed with ethyl alcohol, dried under the vacuum, added into the autoclave, and performed with the previous reaction conditions. Noticeable decline in catalytic activity of RhP_2_/LC_400_ was unobserved during the consecutive seven-time recycling (Figure 7D). Our XRD, XPS, and TEM analyses of the recovered RhP_2_/LC_400_ indicated its high durability (Figure S5). However, the recovered RhP_2_/LC_400_ exhibited an evidently decreased specific surface area when compared with the fresh one (Table 1 and Figure S2), presumably owing to a strong adsorption of **3a** on the RhP_2_/LC_400_ surface. In addition to Rh_2_P/LC_400_, Rh/LC_400_ also showed excellent recyclability and durability during the consecutive seven-time recycling (Figures S6–S8). The above recycling experiments thus demonstrated outstanding stability of lignin-derived Rh catalysts.

### Scope of the hydrogenative coupling

**Scheme 4:**
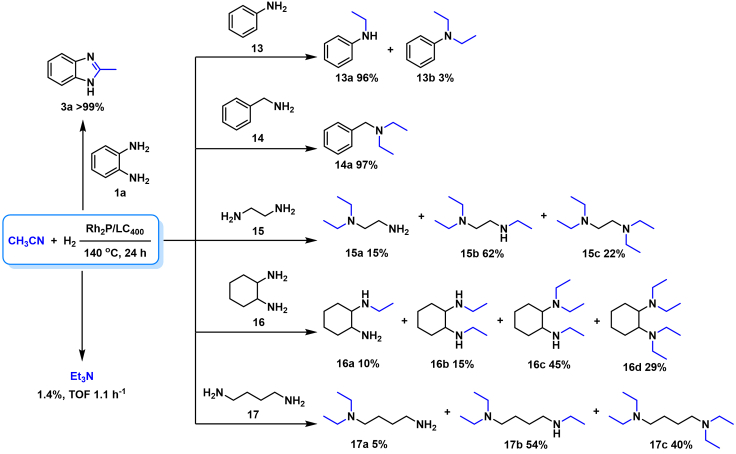
Effect of amines on the hydrogenative coupling Performed with amine (0.3 mmol), Rh_2_P/LC_400_ (20 mg), *P*_H2_ (1.0 MPa), CH_3_CN (3.0 mL).

Therefore, we have demonstrated hydrogenative coupling of **1a**−CH_3_CN for efficient synthesis of **3a** (Scheme 4). Without addition of **1a**, a direct hydrogenation of CH_3_CN afforded trace amount of triethylamine (1.4% yield) with TOF of 1.1 mol_Et3N_ mol_Rh_^−1^ h^−1^ (Scheme 4). To obtain complementary information of the hydrogenative coupling for mechanism investigation, in addition to aromatic 1,2-diamine **1a**, primary monoamines such as aniline (**13**) and benzylamine (**14**) were respectively introduced into the CH_3_CN−hydrogenation system to quickly condense with the *in situ* formed ethanimine intermediate (**6**, R_1_ = CH_3_, Scheme 2B). In the case of **13**, both *N*-ethylaniline (**13a**) and *N*,*N*-diethylaniline (**13b**) were observed under the hydrogenation conditions with secondary amine (**13a**) as the major product (Scheme 4). This result is presumably attributed to weaker nucleophilicity and steric hindrance of the resulting aromatic amine **13a**, which inhibits its further reductive amination with CH_3_CN. As expected, tertiary amine of *N*-benzyl-*N*-ethylethanamine (**14a**, Scheme 4) was obtained in a quantitative yield from **14** by consecutive ethylation. Finally, various aliphatic diamines such as ethylenediamine (**15**), 1,2-cyclohexanediamine (**16**), and 1,4-butanediamine (**17**) were further probed with the CH_3_CN−hydrogenation system. In the cases of **15** and **16**, cyclization products of hydro-1*H*-imidazoles were unobserved, the CH_3_CN−hydrogenation system directly followed ethylation pathway under the investigated conditions by yielding a mixture of stepwisely ethylated amines products (Scheme 4). For long chain aliphatic diamine **17**, similar results were observed with various ethyl-substituted amines as products. Therefore, CH_3_CN functions as ethylation reagent for aromatic monoamine (**13**), aliphatic monoamine (**14**), and aliphatic diamine (**15**–**17**) *via* the reductive substitution reaction. Our research thus demonstrated that Rh_2_P/LC_400_ is a versatile and efficient catalyst by hydrogenative coupling of CH_3_CN with aromatic 1,2-diamine to afford benzimidazole (Scheme 4). While, hydrogenative coupling of CH_3_CN with monoamine and aliphatic diamine led to formation ethyl-substituted amine. Finally, a direct hydrogenation of CH_3_CN with Rh_2_P/LC_400_ yielded trace amount of triethylamine.

**Figure 8:**
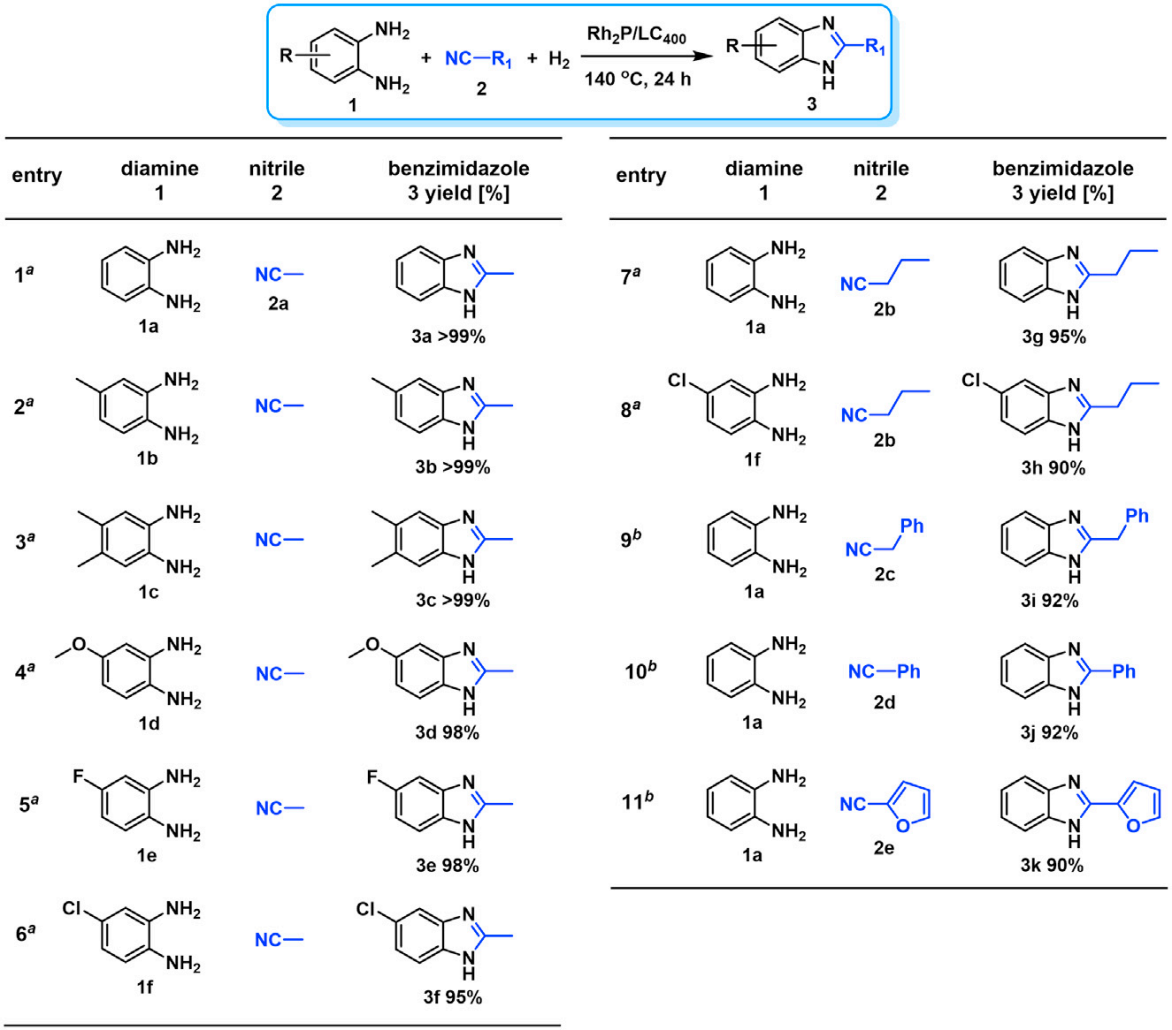
Scope of diamines and nitriles for hydrogenative coupling ^*a*^Performed with Rh_2_P/LC_400_ (20 mg), **1** (0.3 mmol), **2** (3.0 mL), *P*_H2_ (1.0 MPa). ^*b*^**2** (3.0 mmol) in THF (3.0 mL) was used instead of **2** (3.0 mL).

The coupling scope was then evaluated by using Rh_2_P/LC_400_ for a variety of substituted **1a** with CH_3_CN under the optimal conditions (Figure 8). Various substituted **1a** were readily coupled with CH_3_CN to give the corresponding benzimidazoles (**3a−f**) in excellent yields. The substituted **1a** bearing electron-donating groups (CH_3_, OCH_3_; **1b−d**) and electron-withdrawing groups (F, Cl; **1e−f**) were all selectively and quantitatively transformed into the corresponding **3** with the current catalytic system. Notably, in the case of 4-chloro-1,2-phenylenediamine (**1f**), dehalogenation products were unobserved. In addition to CH_3_CN, various nitriles such as *n*-butylnitrile (**2b**), phenylacetonitrile (**2c**), benzonitrile (**2d**), and 2-furonitrile (**2e**) were also applicable to the hydrogenative coupling system by affording excellent yields of the corresponding 2-alkylbenzimidazoles (**3g−k**). THF solvent was used when the reductive couplings were performed with **2c−e**. The above results thus demonstrated that the developed Rh_2_P/LC_400_ catalyst enables highly selective and efficient synthesis of various 2-alkylbenzimidazoles by the hydrogenative coupling of the corresponding nitriles and aromatic 1,2-diamines (Figures S10–S20). In addition to 2-alkylbenzimidazoles, 2-unsubstituted benzimidazoles were recently reported in 35–86% yields with a wide tolerance of functional groups by a direct coupling of **1** with dimethyl sulfoxide (DMSO), in which DMSO functions as methyne source, oxidant, and solvent (Zhu et al., 2020). These two synthetic methods thus highlight a powerful and important extension to benzimidazoles in a sustainable and green way.

### Proposed reaction mechanism

Finally, Scheme 2B shows proposed hydrogenative coupling mechanism with Rh_2_P/LC_400_ catalyst for **3** formation. Hydrogenation (with H_2_) and transfer hydrogenation (with N_2_H_4_) of nitrile **2** lead to formation imine intermediate **6** and primary amine **7**. Subsequent reaction of **1** with **6** results in **11** with elimination of NH_3_. Cyclization of **11** followed by catalytic dehydrogenation of intermediate **12** give **3** with aromatization as a driven force. Moreover, **7** reacts with **6** leading to the formation of secondary imine **8** with exclusion of NH_3_. A successive reaction of **8** with **1** also yields **11** with release of **7**. Notably, an equivalent amount of H_2_ to nitrile molecule is required to initialize the reaction. However, an equivalent H_2_ is finally released from dehydrogenative aromatization step for **3** formation. Therefore, “hydrogen-borrowing” (HB) mechanism is involved in the coupling reaction. The reductive coupling reaction is thus initialized from nitrile-activation by hydrogenation.

**Scheme 5:**
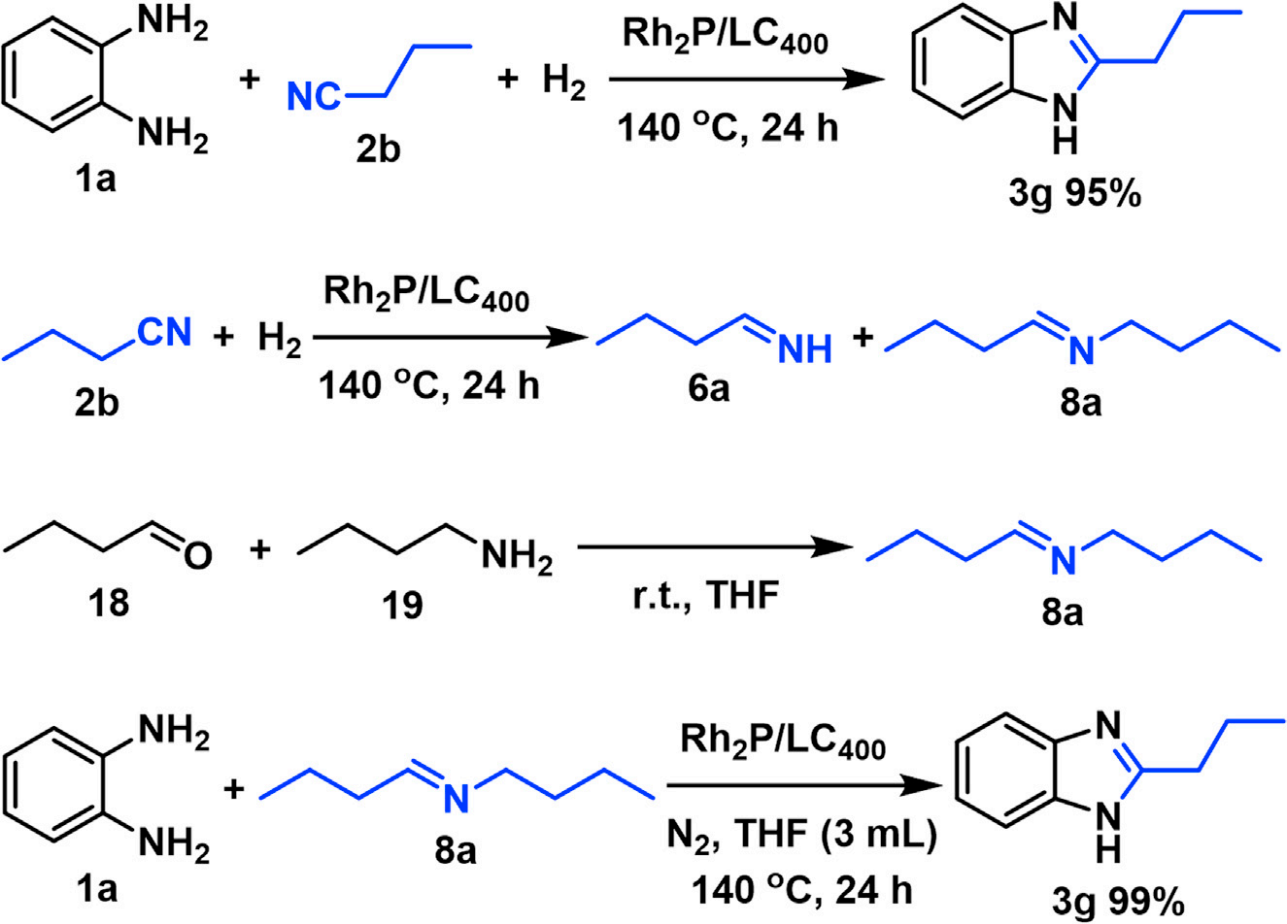
Controlled experiments for mechanism investigation

Controlled experiments were then performed for mechanism investigation (Scheme 5). Our coupling scope experiments have demonstrated that hydrogenative coupling of **2b** with **1a** led to 2-propyl-1*H*-benzo[*d*]imidazole (**3g**) formation in excellent yield. According to above proposed mechanism (Scheme 2B), the reductive coupling process should be initialized from catalytic hydrogenation of **2b** with butan-1-imine (**6a**) and *N*-butylbutan-1-imine (**8a**) as the intermediates (Scheme 5). Due to high reactivity of **6a**, **8a** (Figure S9) was instead directly prepared by condensation of *n*-butyl aldehyde (**18**) and *n*-butylamine (**19**). As expected, treatment **8a** with **1a** in the presence of Rh_2_P/LC_400_ quantitatively afforded **3g** under N_2_ atmosphere in THF, thus demonstrating intermediate nature of **8a**. Rh_2_P/LC_400_ mainly promoted catalytic dehydrogenation process for aromatization under such conditions. Our controlled experiments thus suggested the mechanism as shown in Scheme 2B.

In summary, a direct reductive coupling of nitriles and 1,2-phenylenediamines was readily achieved with Rh_2_P catalyst to give various benzimidazoles in excellent yields (95%–99%). Both H_2_ and N_2_H_4_⋅H_2_O are effective hydrogen sources for the reductive coupling reaction. The high efficiency of Rh_2_P in the reaction is presumably attributed to the enhanced charge transfer to Rh, as well as strong interactions between P and Rh. Isotope trace experiments confirmed the presence of H/D exchange in reductive coupling between H_2_ and the inert –CD_3_ group of CD_3_CN *via* an intramolecular D-shift. The Rh_2_P catalyst can be easily recycled at least for seven times without any significant deactivations. This efficient and convenient method should have great impact on the synthesis of benzimidazole-based heterocyclic compounds.

### Limitations of the study

The developed reductive coupling reaction works well with various 1,2-diamines and nitriles to give the corresponding benzimidazoles. However, the hydrogenative coupling of 3,4-diaminobenzonitrile with CH_3_CN led to formation a serial of products with full conversion of 3,4-diaminobenzonitrile, suggesting a complicated competition between hydrogenation, hydrogenolysis, ethylation and consecutive ethylation. Direct co-coupling of 3,4-diaminobenzonitrile was unobserved in THF solvent in the absence of CH_3_CN. The detailed reason is still not clear.

## STAR★Methods

### Key resources table

**Table:** 

REAGENT or RESOURCE	SOURCE	IDENTIFIER
**Chemicals, peptides, and recombinant proteins**		

RhCl_3_⋅xH_2_O	Aladdin Co. Ltd.	CAS 20765-98-4
PdCl_2_	Aladdin Co. Ltd.	CAS 7647-10-1
RuCl_3_⋅xH_2_O	Aladdin Co. Ltd.	CAS 14898-67-0
N_2_H_4_⋅H_2_O	Aladdin Co. Ltd.	CAS 7803-57-8
Aniline	Aladdin Co. Ltd.	CAS 62-53-3
Butyronitrile	Aladdin Co. Ltd.	CAS 109-74-0
Phenylacetonitrile	Aladdin Co. Ltd.	CAS 140-29-4
Benzonitrile	Aladdin Co. Ltd.	CAS 100-47-0
2-Furonitrile	Aladdin Co. Ltd.	CAS 617-90-3
Benzylamine	Aladdin Co. Ltd.	CAS 100-46-9
Ethylenediamine	Aladdin Co. Ltd.	CAS 107-15-3
1,2-Diaminocyclohexane	Aladdin Co. Ltd.	CAS 694-83-7
1,4-Diaminobutane	Aladdin Co. Ltd.	CAS 110-60-1
Butylamine	Aladdin Co. Ltd.	CAS 109-73-9
Butyraldehyde	Aladdin Co. Ltd.	CAS 123-72-8
Acetonitrile	Guangzhou Kutai Trade Co. Ltd	CAS 75-05-8
Tetrahydrofuran	Guangzhou Kutai Trade Co. Ltd	CAS 109-99-9
High purity gases H_2_(≥99.9%)	Guangzhou Yinglai Gas Co. Ltd.	CAS 1333-74-0
High purity gases N_2_(≥99.9%)	Guangzhou Yinglai Gas Co. Ltd.	CAS 7727-37-9
1,2-Phenylenediamine	Macklin Biochemical Science Co. Ltd.	CAS 95-54-5
3,4-Diaminotoluene	Bide Pharmatech Co. Ltd.	CAS 496-72-0
4,5-Dimethylbenzene-1,2-diamine	Bide Pharmatech Co. Ltd.	CAS 3171-45-7
4-Methoxy-o-phenylenediamine	Bide Pharmatech Co. Ltd.	CAS 102-51-2
4-Fluorobenzene-1,2-diamine	Bide Pharmatech Co. Ltd.	CAS 367-31-7
4-Chlorobenzene-1,2-diamine	Bide Pharmatech Co. Ltd.	CAS 95-83-0

**Other**		

Optima 2000 DV inductively coupled plasma atomic emission spectrometer (ICP-AES)	PerkinElmer, USA	https://www.perkinelmer.com.cn
Vertex 70 FI-TR spectrometer	Bruker	https://bruker.com
ULTRA 55 Scanning Electron Microscope (SEM)	Zeiss	http://www.lingrn.com
TECNAL-12 Transmission Electron Microscope (TEM)	FEI	https://www.thermofisher.cn
TriStar II 3flex adsorption analyzer	Micromeritics	https://www.micromeritics.com
D8 ADVANCE X-ray Powder Diffractometer (XRD)	Bruker	https://bruker.com
K-Alpha X-Ray photoelectron spectrometer (XPS)	Thermo scientific	https://www.thermofisher.cn
Fuli 9790 (Type II) Gas Chromatography (GC)	Fuli Instruments	http://www.cnfuli.com.cn
GC-2010 Plus Gas Chromatography-Mass Spectrometer (GC-MS)	Shimadzu Corporation of Japan	https://www.shimadzu.com/
AV III 300 (300 MHz) spectrometer	Bruker	https://bruker.com

### Resource availability

#### Lead contact

Further information and requests for resources and reagents should be directed to and will be fulfilled by the lead contact, Jinzhu Chen (chenjz@jnu.edu.cn).

#### Materials availability

This work did not generate new unique reagents. All stable reagents generated in this study are available from the lead contact without restriction.

### Method details

#### Materials synthesis

*Preparation of Rh(PPh*_*3*_*)*_*3*_*Cl and Rh(dppe)*_*2*_*Cl:* Rh(PPh_3_)_3_Cl and Rh(dppe)_2_Cl were prepared according to literature methods (Osborn and Wilkinson, 1967; Kunin et al., 1985).

*Preparation of LC:* LC was prepared according to the literature method with slight modification (Chen et al., 2020), enzymatic hydrolysis lignin (EHL, 3.0 g) and potassium bicarbonate (6.0 g) were finely grinded and mixed uniformly in a mortar. The resulting mixture was then transferred into an alundum boat and calcined under nitrogen atmosphere at 800°C for 3 h with a heating rate of 2°C min^−1^. The obtained black solid was collected, grinded into black powder, and further leached with aqueous HCl solution (100 mL, 2 mol L^−1^) for 12 h with vigorous stirring at room temperature. LC was then obtained as black solid (about 300 mg) by solution filtration, thoroughly washed with water (1 L), and dried at 80°C under the vacuum overnight.

*Preparation of Rh*_*2*_*P/LC*_*400*_*:* RhCl_3_⋅xH_2_O (25 mg, 0.095 mmol) and 1,2-*bis*(diphenylphosphino)ethane (dppe, 150 mg, 0.38 mmol) were dissolved in tetrahydrofuran (THF, 80 mL) in a round bottom flask. LC (150 mg) was subsequently added into the above solution, the resulting mixture was vigorously stirred at 60°C for 12 h. THF solvent in the mixture was then removed by rotary evaporator under reduced pressure. The obtained mixture was transferred into a quartz boat and calcined under atmospheric mixture of H_2_/N_2_ (V_H2_:V_N2_ = 8%) for 2 h at 400°C with a heating rate of 5°C min^−1^ to give Rh_2_P/LC_400_ (Rh, 4.8 wt.%). The Rh content was obtained by inductively coupled plasma-atomic emission spectrometry (ICP-AES) analysis.

*Preparation of Rh/LC*_*400*_*:* Rh/LC_400_ (Rh, 5.5 wt.%) was prepared with the same procedure used for Rh_2_P/LC_400_ without addition of dppe.

*Preparation of Pd/LC*_*400*_*:* Pd/LC_400_ (Pd, 5.2 wt.%) was prepared with the same procedure used for Rh_2_P/LC_400_ except that RhCl_3_⋅xH_2_O (25 mg) and THF solvent were respectively replaced by PdCl_2_ (25 mg) and acetonitrile solvent.

*Preparation of Ru/LC*_*400*_*:* Ru/LC_400_ (Ru, 5.1 wt.%) was prepared with the same procedure used for Rh_2_P/LC_400_ except that RhCl_3_⋅xH_2_O (25 mg) was replaced by RuCl_3_⋅xH_2_O (25 mg).

#### General procedure

*Reductive coupling:* Typically, Rh_2_P/LC_400_ (20 mg), 1,2-phenylenediamine (**1a**, 32 mg, 0.3 mmol) and acetonitrile (**2a**, 3.0 mL) were added into a Teflon-lined autoclave reactor (25 mL). Then, H_2_ (0.5 MPa) was slowly loaded into the reactor to remove the air inside for three times. Finally, H_2_ (1.0 MPa) was slowly charged into the reactor at ambient temperature. The reductive coupling was performed for 24 h at 140°C. After the reaction, the obtained mixture was analyzed by Gas Chromatography.

*Calculation of****1a****conversion and****3a****yield:* After the reaction, the mixture was filtered to remove catalyst, the resulting filtrate was then transferred into a volumetric flask, diluted to the volume with acetonitrile, and finally analyzed by GC. **1a** conversion and **3a** yield were then obtained by the corresponding formulas:

$$1a\;conversion\left(\%\right)=\left(\frac{moles\;of\;1a\;converted}{moles\;of\;1a\;fed}\right)\times 100$$

$$3a\;yield\left(\%\right)=\left(\frac{moles\;of\;3a\;produced}{moles\;of\;1a\;fed}\right)\times 100$$

#### Characterization of products 3a-3k (NMR data)

##### 2-Methyl-1*H*-benzo[*d*]imidazole **3a**

**Figure:**
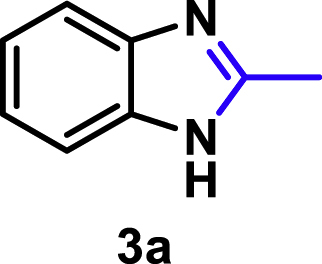

Yield: 39.3 mg (99%). ^1^H NMR (300 MHz, CD_3_CN, 25°C) δ 7.48 (dd, *J* = 6.0, 3.2 Hz, 2H), 7.15 (dd, *J* = 6.0, 3.2 Hz, 2H), 2.52 (s, 3H). ^13^C {^1^H} NMR (75 MHz, CDCl_3_, 25°C) δ = 151.28, 138.77, 122.32, 114.63, 15.13 ppm. The spectral data is consistent with the literature data (, 2020b).

##### 2,5-Dimethyl-1*H*-benzo[*d*]imidazole. **3b**

**Figure:**
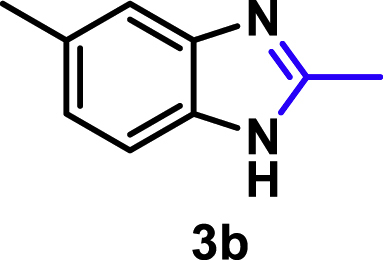

Yield: 43.5 mg (99%). ^1^H NMR (300 MHz, DMSO-*d*_6_, 25°C) δ 7.35 (d, *J* = 8.1 Hz, 1H), 7.25 (s, 1H), 6.95 (d, *J* = 8.1 Hz, 1H), 2.47 (s, 3H), 2.38 (s, 3H). ^13^C {^1^H} NMR (75 MHz, DMSO, 25°C) δ 150.81, 138.19, 136.79, 130.35, 122.65, 114.01, 113.63, 21.30, 14.40. The spectral data is consistent with the literature data (Gan et al., 2018).

##### 2,5,6-Trimethyl-1*H*-benzo[*d*]imidazole. **3c**

**Figure:**
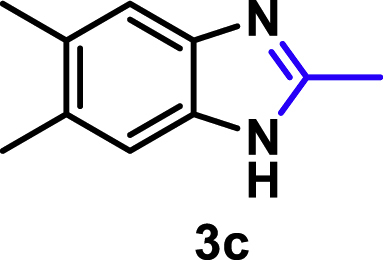

Yield: 47.6 mg (99%). ^1^H NMR (300 MHz, DMSO-*d*_6_, 25°C) δ 7.20 (s, 2H), 2.43 (s, 3H), 2.27 (s, 6H). ^13^C {^1^H} NMR (75 MHz, DMSO-*d*_6_, 25°C) δ 150.16, 129.06, 19.88, 14.57. The spectral data is consistent with the literature data (Gan et al., 2018).

##### 5-Methoxy-2-methyl-1*H*-benzo[*d*]imidazole. **3d**

**Figure:**
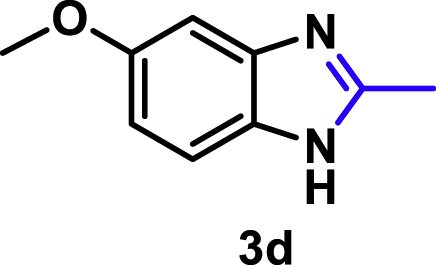

Yield: 47.7 mg (98%). ^1^H NMR (300 MHz, DMSO-*d*_6_, 25°C) δ 7.33 (d, *J* = 8.6 Hz, 1H), 6.96 (s, 1H), 6.74 (d, *J* = 10.7 Hz, 1H), 3.75 (s, 3H), 2.44 (s, 3H). ^13^C {^1^H} NMR (75 MHz, DMSO-*d*_6_, 25°C) δ 155.08, 150.80, 110.08, 55.41, 14.64. The spectral data is consistent with the literature data (Wan et al., 2019).

##### 5-Fluoro-2-methyl-1*H*-benzo[*d*]imidazole. **3e**

**Figure:**
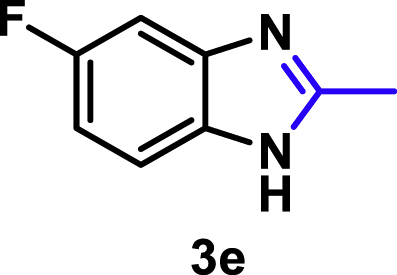

Yield: 44.1 mg (98%). ^1^H NMR (300 MHz, DMSO-*d*_6_, 25°C) δ 7.45 (dd, *J* = 8.6, 4.9 Hz, 1H), 7.27 (d, *J* = 9.6 Hz, 1H), 6.98 (t, *J* = 9.3 Hz, 1H), 2.47 (s, 3H). ^13^C {^1^H} NMR (75 MHz, DMSO-*d*_6_, 25°C) δ 159.69, 156.59, 152.81, 109.00, 108.67, 40.80, 14.65. The spectral data is consistent with the literature data (Yamini et al., 2021).

##### 5-Chloro-2-methyl-1*H*-benzo[*d*]imidazole. **3f**

**Figure:**
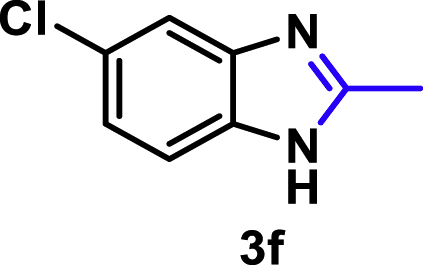

Yield: 47.5 mg (95%). ^1^H NMR (300 MHz, DMSO-*d*_6_, 25°C) δ 7.50 (s, 1H), 7.45 (d, *J* = 8.5 Hz, 1H), 7.14 (d, *J* = 8.5 Hz, 1H), 2.48 (s, 3H). ^13^C {^1^H} NMR (75 MHz, DMSO-*d*_6_, 25°C) δ 153.00, 125.48, 121.25, 115.13, 114.22, 14.63. The spectral data is consistent with the literature data (Gan et al., 2018).

##### 2-Propyl-1*H*-benzo[*d*]imidazole. **3g**

**Figure:**
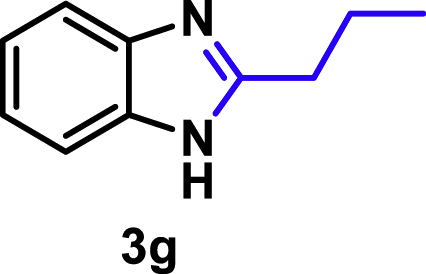

Yield: 45.6 mg (95%). ^1^H NMR (300 MHz, DMSO-*d*_6_, 25°C) δ 12.18 (s, 1H), 7.45 (s, 2H), 7.09 (d, *J* = 5.9 Hz, 2H), 2.78 (t, *J* = 7.4 Hz, 2H), 1.75 (dd, *J* = 14.8, 7.4 Hz, 2H), 0.94 (t, *J* = 7.4 Hz, 3H). ^13^C {^1^H} NMR (75 MHz, DMSO-*d*_6_, 25°C) δ 155.05, 121.04, 30.56, 21.03, 13.71. The spectral data is consistent with the literature data (An et al., 2021).

##### 5-Chloro-2-propyl-1*H*-benzo[*d*]imidazole. **3h**

**Figure:**
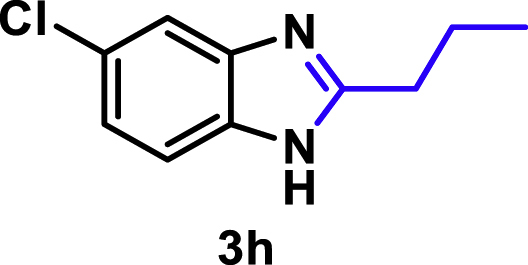

Yield: 52.4 mg (90%). ^1^H NMR (300 MHz, DMSO-*d*_6_) δ 12.36 (s, 1H), 7.73–7.27 (m, 2H), 7.12 (s, 1H), 2.77 (t, *J* = 7.2 Hz, 2H), 1.77 (q, *J* = 7.3 Hz, 2H), 0.93 (t, *J* = 7.2 Hz, 3H). ^13^C {^1^H} NMR (75 MHz, DMSO-*d*_6_, 25°C) δ 156.65, 125.20, 121.24, 115.26, 30.90, 20.89, 13.18. The spectral data is consistent with the literature data (Sriramoju et al., 2018).

##### 2-Benzyl-1*H*-benzo[*d*]imidazole. **3i**

**Figure:**
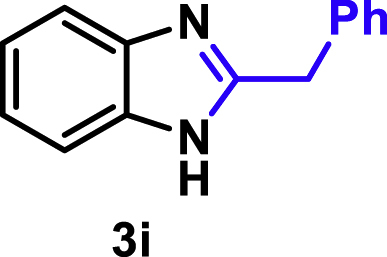

Yield: 57.4 mg (92%). ^1^H NMR (300 MHz, DMSO-*d*_6_) δ 12.25 (s, 1H), 7.47 (dd, *J* = 5.8, 3.2 Hz, 2H), 7.39–7.26 (m, 4H), 7.23 (dd, *J* = 3.4, 2.4 Hz, 1H), 7.12 (dd, *J* = 5.9, 3.1 Hz, 2H), 4.17 (s, 2H). ^13^C{^1^H} NMR (75 MHz, DMSO-*d*_6_) δ 153.68, 139.48, 137.10, 128.83, 126.58, 121.43, 114.71, 35.08. The spectral data is consistent with the literature data (An et al., 2021).

##### 2-Phenyl-1*H*-benzo[*d*]imidazole. **3j**

**Figure:**
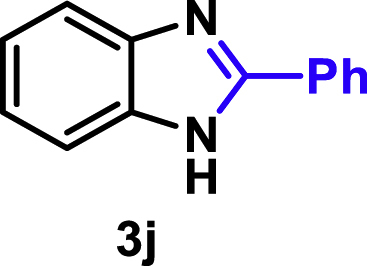

Yield: 53.5 mg (92%). ^1^H NMR (300 MHz, 25°C, DMSO-*d*_6_) δ 12.95 (s, 1H), 8.20 (d, *J* = 8.2 Hz, 2H), 7.89–7.38 (m, 5H), 7.35–7.01 (m, 2H). ^13^C{^1^H} NMR (75 MHz, 25°C, DMSO-*d*_6_) δ 151.25, 130.19, 129.87, 128.99, 126.46, 122.14. The spectral data is consistent with the literature data (Lin et al., 2021).

##### 2-(2-furanyl)-1*H*-benzo[*d*]imidazole. **3k**

**Figure:**
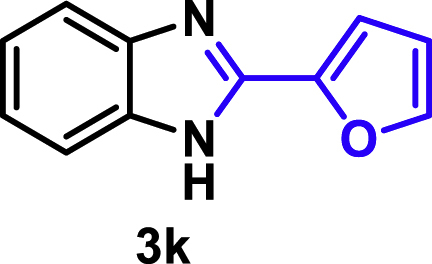

Yield: 49.7 mg (90%). ^1^H NMR (300 MHz, 25°C, DMSO-*d*_6_) δ 12.93 (s, 1H), 7.91 (d, *J* = 18.7 Hz, 1H), 7.54 (s, 2H), 7.18 (d, *J* = 16.8 Hz, 3H), 6.69 (d, *J* = 19.4 Hz, 1H). ^13^C{^1^H} NMR (101 MHz, 25°C, DMSO-*d*_6_) δ 145.45, 144.61, 143.55, 134.19, 122.46, 121.81, 118.72, 112.30, 111.34, 110.44. The spectral data is consistent with the literature data (Lin et al., 2021).

## Data Availability

Original code was not used in this manuscript. All relevant data supporting the findings of this study are available within the paper and its Supplemental Information files. Any additional information required to reanalyze the data reported in this paper is available from the lead contact upon reasonable request.
