# Supplementary material for: Hydrogenative coupling of nitriles with diamines to benzimidazoles using lignin-derived Rh2P catalyst
Source: iScience. 2021 Aug 28;24(9):103045. doi: 10.1016/j.isci.2021.103045 (PMC8450259; doi:10.1016/j.isci.2021.103045)
Supplement: Document S1. Figures S1–S20 and Tables S1–S8 [file mmc1.pdf]

iScience, Volume *24*

Supplemental information

Hydrogenative coupling of nitriles with diamines to benzimidazoles using lignin-derived Rh_2P catalyst

Jiarui Zhang, Ruxu Yao, Jinzhu Chen, Tao Li, and Yisheng Xu

Table S1. Various catalytic system for acetonitrile hydrogenation to amine. Related to Scheme 4.

Catalyst	Reaction	Reaction Conditions
NC-800 (Wang et al., 2019)	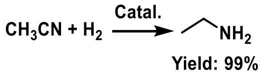	Nitriles (1 mmol), NC-800 (catalyst 20 mg), solvent (DMSO: deionized water = 1:0.5) (1.5 mL), $NaBH_4$ (5 mmol), 60 °C, 4.5 h.
Pd-$Cu_{0.5}$/Fe_3O_4 (Liu et al., 2018)	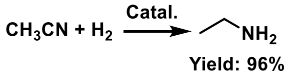	Nitrile (1 mmol), $H_3N{\cdot}BH_3$ (3 mmol), catalyst (10 wt.% of substrate), CH_3OH (2 mL), 40 °C, 90 min.
Pd (2.56 wt%) supported on CB (Nandi et al., 2017)	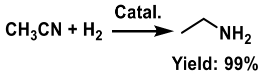	Nitriles (42 mmol), catalyst (1 wt% w.r.t substrate), H_2O (10 mL), H_2 (10 bar), stirrer speed (1000 rpm), 80 °C, 3 h.
Cp*Mo(1,2-$Ph_2PC_6H_4S$)(η^2-NCMe) (Hou et al., 2020)	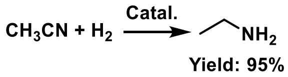	Nitriles (0.5 mmol), $H_3N{\cdot}BH_3$ (1 mmol), catalyst (2 mol %), THF (1 mL), RT, 8 h.
[Pd(C_6H_4CH=N–P)(PhCN)Cl] (P = poly N-vinylcarbazole) (Islam et al., 2010)	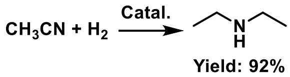	Nitriles (0.5 mol), catalyst (1.20 mmol L^{-1}), H_2 (50 bar), 90 °C, 5.5 h.
Pd-$Fe_{0.25}Cu_{0.25}$/Fe_3O_4 (Liu et al., 2018)	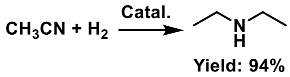	Nitrile (1 mmol), $H_3N{\cdot}BH_3$ (3 mmol), catalyst (10 wt.% of substrate), CH_3OH (2 mL), 40 °C, 90 min.
Pd@mpg-C_3N_4 (Li et al., 2012)	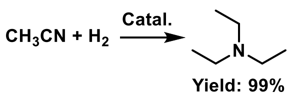	Nitrile (10 mmol), catalyst (0.2% mol Pd relative to substrate), H_2 (1.0 MPa), 70 °C, 6 h.
Rh_2P/LC_{400} (This work)	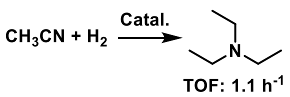	Nitrile (3 mL), catalyst (20 mg), H_2 (1.0 MPa), 140 °C, 24 h.

Table S2. Various methods for 2-methylbenzimidazole synthesis. Related to Schemes 1 and 2.

Catalyst	Reaction	Reaction Conditions
concentrated HCl (Hölljes and Wagner, 1944)	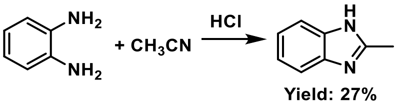	*o*-phenylenediamine (0.02 mol), HCl (0.02 mol), 200 °C, 6 h.
Rh_2P/LC_{400} (This work)	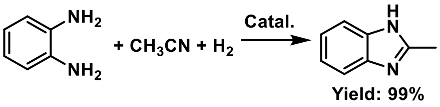	*o*-phenylenediamine (0.3 mmol), catalyst (20 mg), H_2 (1.0 MPa), 140 °C, 24 h.
Rh_2P/LC_{400} (This work)	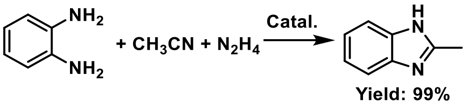	*o*-phenylenediamine (0.3 mmol), catalyst (20 mg), $N_2H_4.H_2O$, N_2 (1.0 MPa), 140 °C, 6 h.
Cu–Pd/γ-Al_2O_3 (Feng et al., 2016)	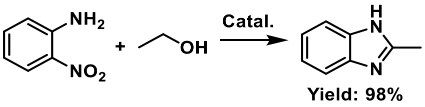	*o*-nitroaniline (6 g), catalyst (1 g), ethanol (100 mL), H_2O (50 mL), N_2 (3.5 MPa), stirrer speed 900 rpm, 180 °C, 12 h.
CdSe/MMT (MMT: montmorillonite) (Wade et al., 2015)	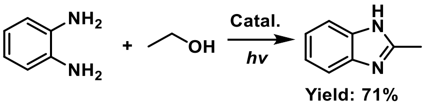	*o*-phenylenediamine (10 mmol), 10% CdSe/MMT (5 mg), acetaldehyde (10 mmol), ethanol (30 mL), RT, 2.5 h, visible light.
C-TiO_2/**1**$_{(600)}$ (Li et al., 2020)	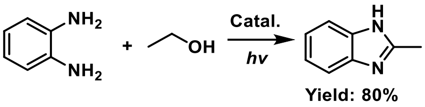	*o*-phenylenediamine (0.1 mmol), catalyst (15 mg), EtOH (4.0 mL), RT, 8 h, visible light irradiation (Xe lamp, λ > 420 nm).
Pt(0.2)@TiO_2 (Shiraishi et al., 2010)	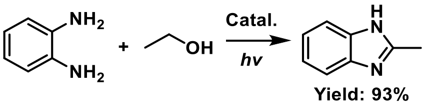	*o*-phenylenediamine (0.1 mmol), catalyst (10 mg), alcohol (10 mL), N_2 (0.1 MPa), 30 °C, 4 h, λ > 300 nm.
C-TiO_2/**1**$_{(600)}$ (Li et al., 2020)	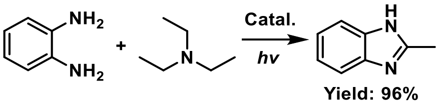	*o*-phenylenediamine (0.1 mmol), catalyst (15 mg), Et_3N (1.0 mL), CH_3CN (3.0 mL), H_2O (0.6 mmol), 100 °C, 14 h, visible light irradiation.
TiO_2 (Wang et al., 1997)	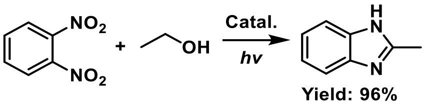	1,2-dinitrobenzene (0.005 M), catalyst (1.0 g/L), alcohol (2.0 mL), RT, 15-30 min, UV light.
TiO_2-P25 (Selvam et al., 2009)	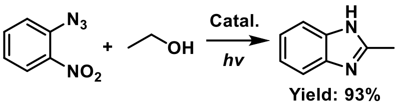	2-nitrophenyl azide (1×10^{-4} M), alcohol (25 mL), catalyst (1.0 g L^{-1}), 32 °C, 4 h, UV light.
Ag-TiO_2 (Selvam et al., 2009)	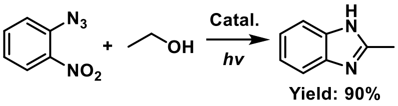	2-nitrophenyl azide (1×10^{-4} M), alcohol (25 mL), catalyst (1.0 g L^{-1}), 32 °C, 4 h, UV light.
Pt-TiO_2 (Selvam et al., 2009)	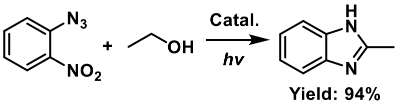	2-nitrophenyl azide (1×10^{-4} M), alcohol (25 mL), catalyst (1.0 g L^{-1}), 32 °C, 4 h, UV light.

Table S3. High-resolution Rh 3d XPS data of Rh catalysts. Related to Figure 4.

Sample	Total Rh Ratio [wt.%]	Rh $3d_{3/2}$ BE [eV]	Rh $3d_{3/2}$ Ratio [%]	Rh $3d_{5/2}$ BE [eV]	Rh $3d_{5/2}$ Ratio [%]	Rh species	Relative content [%]
Rh_2P/LC_{400}	4.8	314.8	12.1	309.8	15.1	Rh^{3+}	27.2
		312.4	27.9	307.7	44.9	Rh_2P	72.8
Rh_2P/LC_{600}	5.2	314.8	11.4	309.8	18.5	Rh^{3+}	29.9
		312.4	27.6	307.7	42.5	Rh_2P	70.1
Rh_2P/LC_{800}	6.5	314.8	11.2	309.8	18.8	Rh^{3+}	30.0
		312.4	28.8	307.7	41.2	Rh_2P	70.0
Rh/LC_{400}	4.6	314.7	11.2	309.7	18.6	Rh^{3+}	29.8
		312.3	26.8	307.6	43.4	Rh	70.2
recovered Rh_2P/LC_{400}	4.1	314.8	13.3	309.8	21.7	Rh^{3+}	35.0
		312.4	26.3	307.7	38.7	Rh_2P	65.0
recovered Rh/LC_{400}	4.2	314.7	10.3	309.7	18.7	Rh^{3+}	29.0
		312.3	28.8	307.6	42.2	Rh	71.0

Table S4. High-resolution Pd 3d XPS data of Pd/LC_{400}. Related to Figure 4.

Sample	Total Pd Ratio [wt.%]	Pd $3d_{3/2}$ BE [eV]	Pd $3d_{3/2}$ Ratio [%]	Pd $3d_{5/2}$ BE [eV]	Pd $3d_{5/2}$ Ratio [%]	Pd species	Relative content [%]
Pd/LC_{400}	6.5	342.2	9.4	337.0	14.2	Pd^{2+}	23.6
		341.2	27.4	336.0	49.0	Pd	76.4

Table S5. High-resolution Ru 3p XPS data of Ru/LC_{400}. Related to Figure 4.

Sample	Total Ru Ratio [wt.%]	Ru $3p_{1/2}$ BE [eV]	Ru $3p_{1/2}$ Ratio [%]	Ru $3p_{3/2}$ BE [eV]	Ru $3p_{3/2}$ Ratio [%]	Ru species	Relative content [%]
Ru/LC_{400}	6.6	486.5	8.5	464.0	15.0	Ru^{3+}	23.5
		484.0	28.3	461.5	48.2	Ru	76.5

Table S6. High-resolution P $2p_{3/2}$ XPS data of various investigated catalysts. Related to Figure 4.

Sample	Total P	P-O		P-C		Rh-P (P $2p_{1/2}$)		Rh-P	
	Ratio [%]	BE [eV]	Ratio [%]	BE [eV]	Ratio [%]	BE [eV]	Ratio [%]	BE [eV]	Ratio [%]
Rh_2P/LC_{400}	5.0	134.5	66.0	133.3	28.0	131.0	-	130.1	6.0
Rh_2P/LC_{600}	4.8	134.9	80.3	133.3	14.2	131.0	-	130.1	5.5
Rh_2P/LC_{800}	5.8	134.3	80.1	133.3	13.1	131.0	-	130.1	6.8
Pd/LC_{400}	7.8	134.5	69.8	133.3	30.2	-	-	-	-
Ru/LC_{400}	4.7	134.5	66.9	133.3	33.1	-	-	-	-
recovered Rh_2P/LC_{400}	4.5	134.3	63.6	133.2	30.0	131.1	-	130.2	6.4

Table S7. High-resolution C 1s XPS data of various investigated catalysts. Related to Figure 4.

Sample	Total C	O=C-O		C=O		C-O/C-P		C-C	
	Ratio [%]	BE [eV]	Ratio [%]	BE [eV]	Ratio [%]	BE [eV]	Ratio [%]	BE [eV]	Ratio [%]
Rh/LC_{400}	83.6	288.8	6.8	287.1	9.5	286.0	12.1	284.6	71.6
Rh_2P/LC_{400}	74.1	288.8	5.7	287.1	6.6	286.0	13.0	284.6	74.7
Rh_2P/LC_{600}	71.3	288.8	5.5	287.1	6.8	286.0	13.3	284.6	74.4
Rh_2P/LC_{800}	69.7	288.8	5.2	287.1	7.1	286.0	12.8	284.6	74.9
Pd/LC_{400}	62.4	288.8	7.4	287.1	8.0	286.0	12.5	284.6	72.1
Ru/LC_{400}	81.5	288.8	7.3	287.1	8.3	286.0	12.5	284.6	71.9
recovered Rh_2P/LC_{400}	87.3	288.8	7.2	287.1	8.1	286.0	12.7	284.6	72.0
recovered Rh/LC_{400}	87.6	288.8	7.8	287.1	10.5	286.0	11.3	284.6	70.4

Table S8. Effect of reaction solvent on the hydrogenative coupling of 1a with 2a for 3a formation. Related to Figure 5.

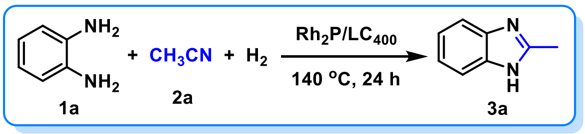

Solvent	**3a** Yield [%]
THF	92%
1,4-dioxane	75%
benzene	54%
dimethyl sulfoxide	14%
dichloromethane	trace

Reaction conditions: Rh_2P/LC_{400} (20 mg), **1a** (0.3 mmol), **2a** (3.0 mmol), P_{H2} (1.0 MPa), solvent (3.0 mL), *t* (24 h), *T* (140 °C).

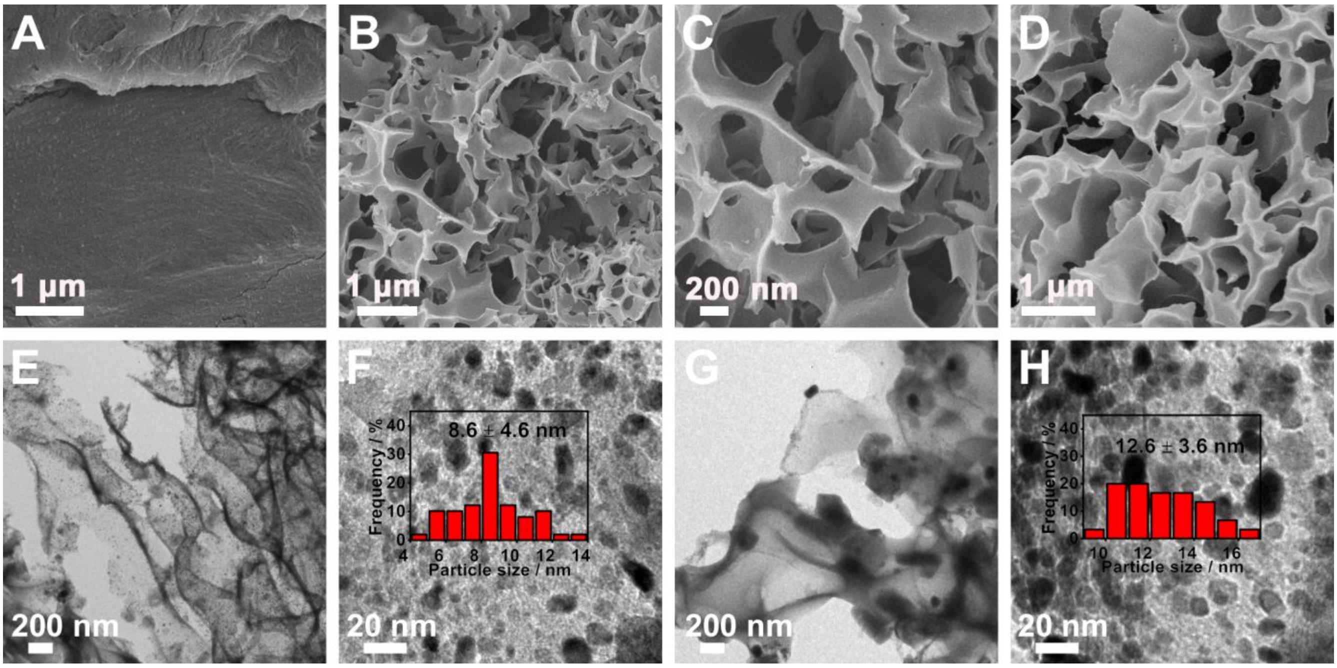

Figure S1. SEM and TEM characterization. SEM: (A) EHL, (B-C) LC, (D) Rh_2P/LC_{400}. TEM: (E-F) Rh_2P/LC_{600}, and (G-H) Rh_2P/LC_{800}. Inserts of (F) and (H) are the size distribution histogram by statistical analysis of 200 Rh_2P nanoparticles. Related to Figures 2 and 5.

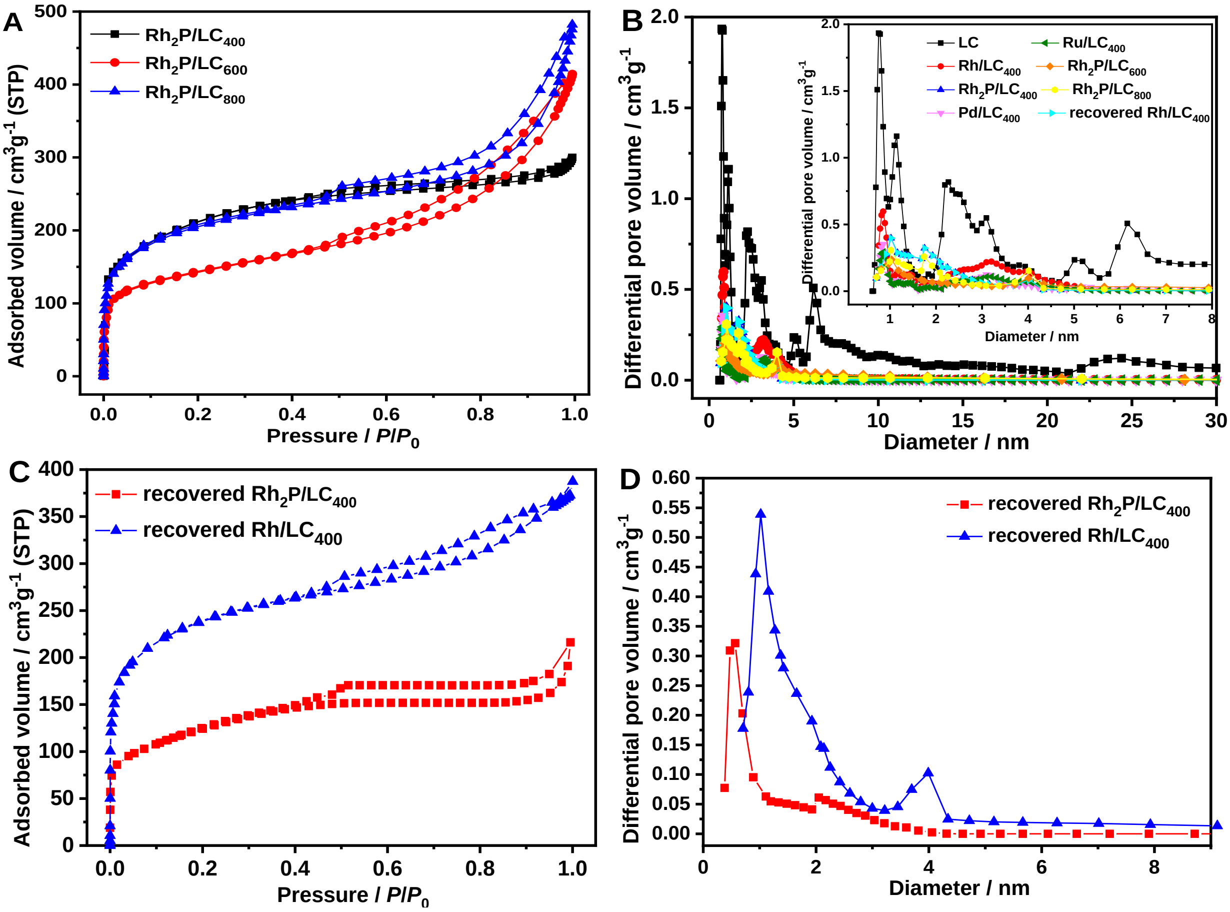

Figure S2. N_2 sorption and pore size distribution analysis. Investigated samples: (A) N_2 sorption isotherm, and (B) pore size distribution. Recovered Rh_2P/LC_{400} and Rh/LC_{400} after a seven-time recycling: (C) N_2 sorption isotherm, and (D) pore size distribution. Related to Table 1, Figures 3 and 7.

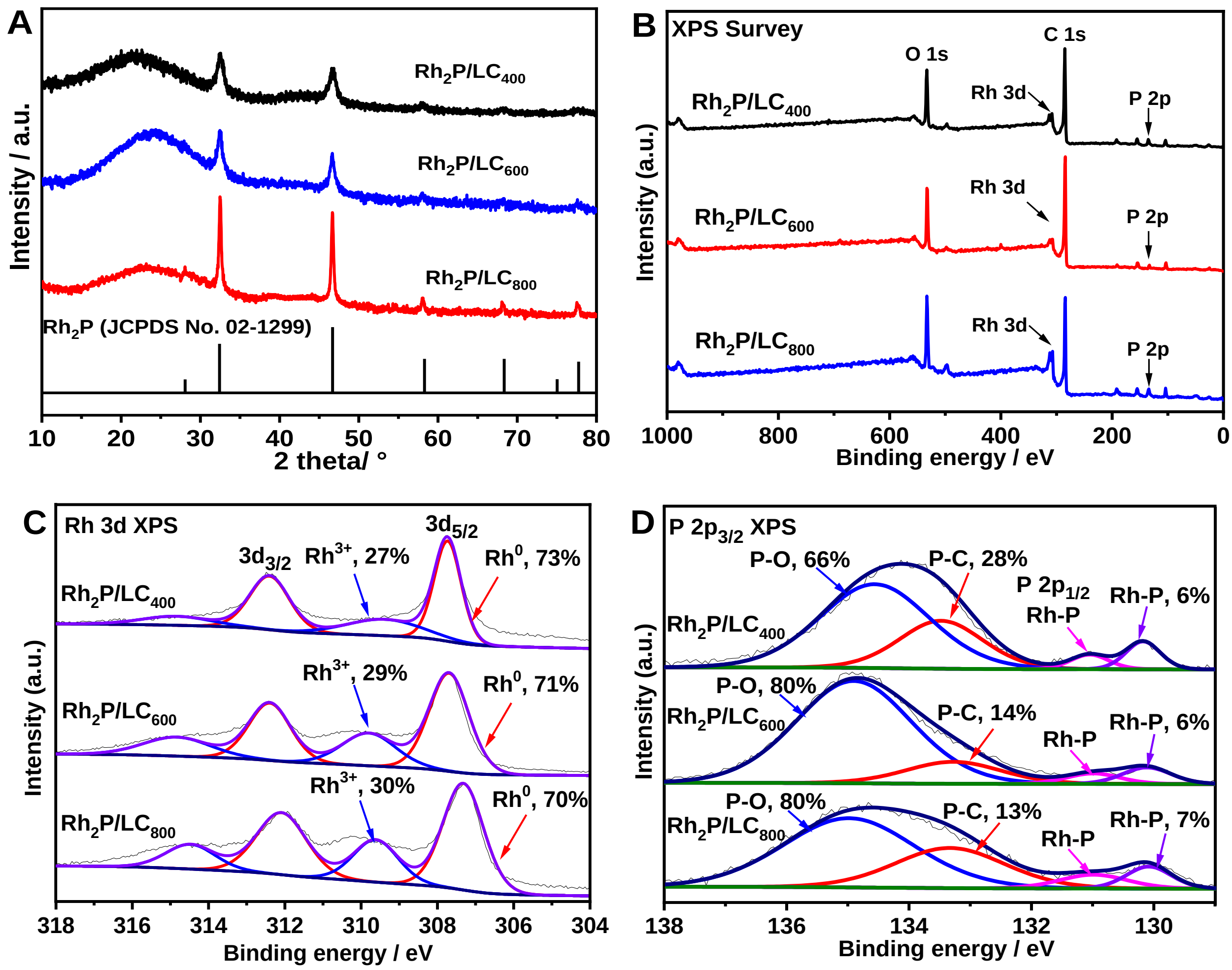

Figure S3. Comparison of Rh_2P/LC_{400}, Rh_2P/LC_{600}, and Rh_2P/LC_{800}: (A) XRD patterns, (B) XPS scan survey, (C) Rh 3d XPS, and (D) P $2p_{3/2}$ XPS. Related to Figure 4.

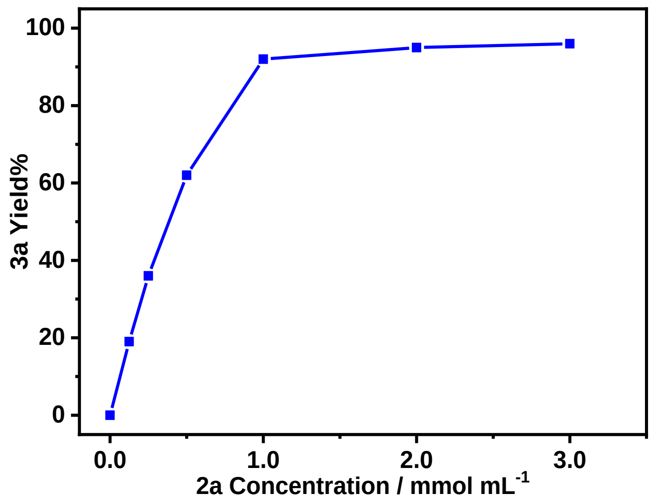

Figure S4. Effect of 2a concentration in THF on the reductive coupling of 1a with 2a. Related to Figure 5. Reaction condition: Rh_2P/LC_{400} (20 mg), **1a** (0.3 mmol), THF (3.0 mL), P_{H2} (1.0 MPa), T (140 °C).

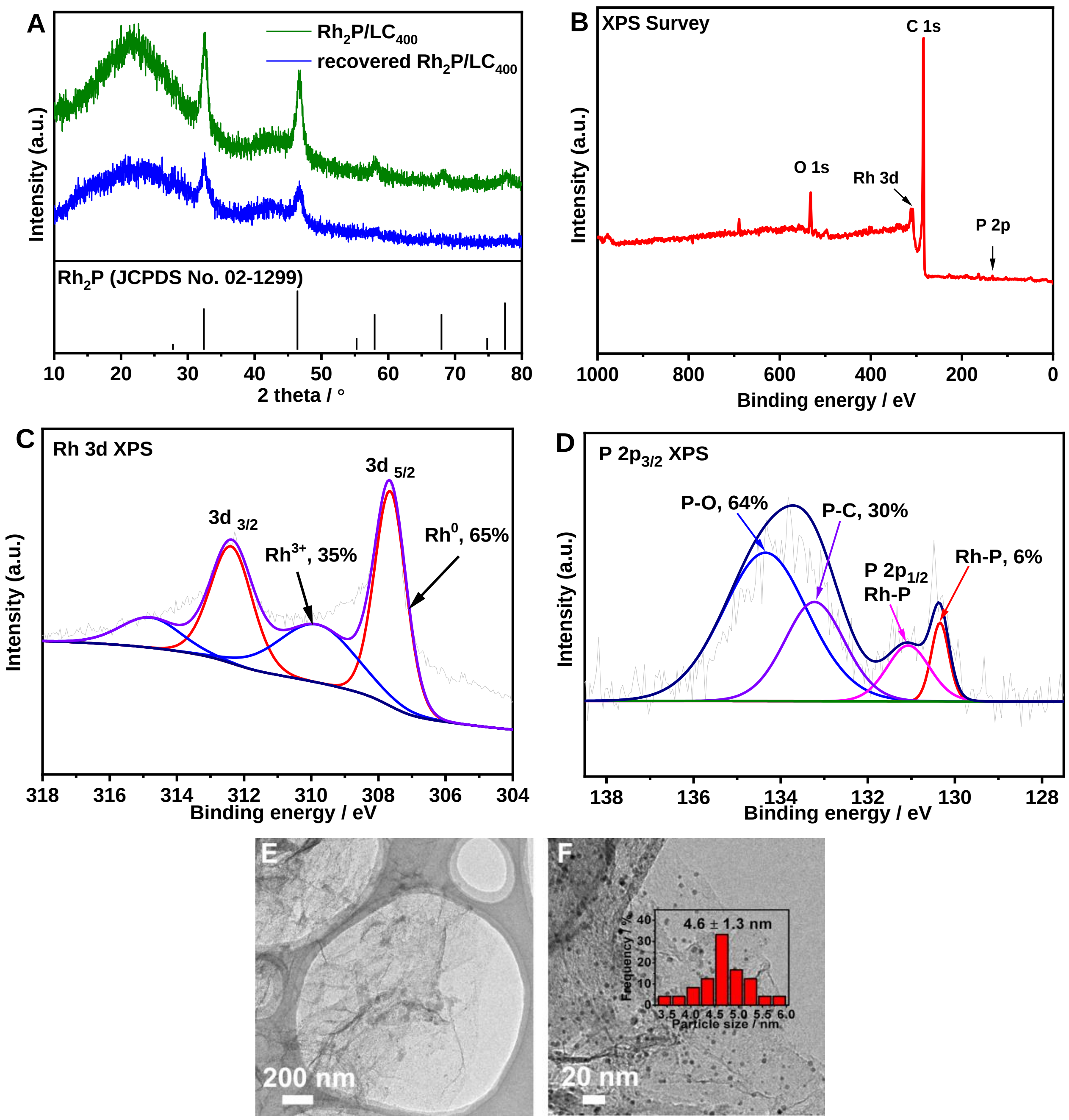

Figure S5. Recovered Rh_2P/LC_{400} after a seven-time recycling: (A) XRD patterns, (B) XPS scan survey, (C) Rh 3d XPS, (D) P $2p_{3/2}$ XPS, and (E-F) TEM images. Insert of (F) is the size distribution histogram by statistical analysis of 200 Rh_2P nanoparticles. Related to Figure 7.

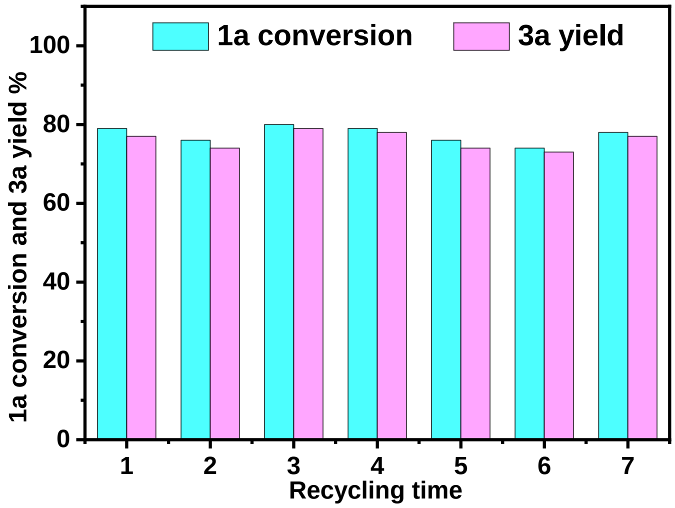

Figure S6. Reusability of Rh/LC$_{400}$. Related to Figure 7. Reaction condition: Rh/LC$_{400}$ (10 mg), **1a** (0.2 mmol), P_{H2} (1.0 MPa), CH_3CN (3.0 mL), *t* (24 h), *T* (140 °C).

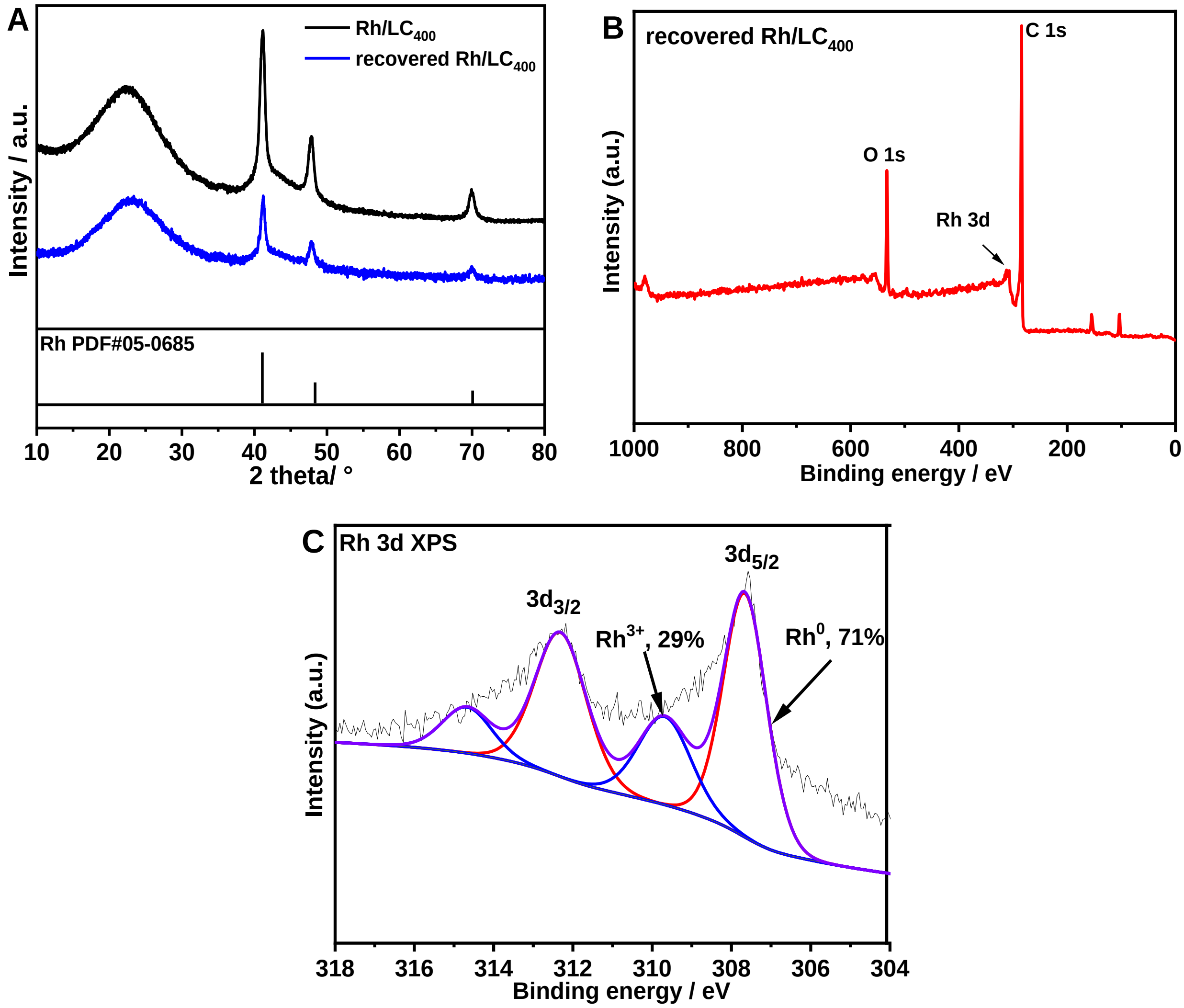

Figure S7. Recovered Rh/LC$_{400}$ after a seven-time recycling: (A) XRD patterns, (B) XPS scan survey, (C) Rh 3d XPS. Related to Figure 7.

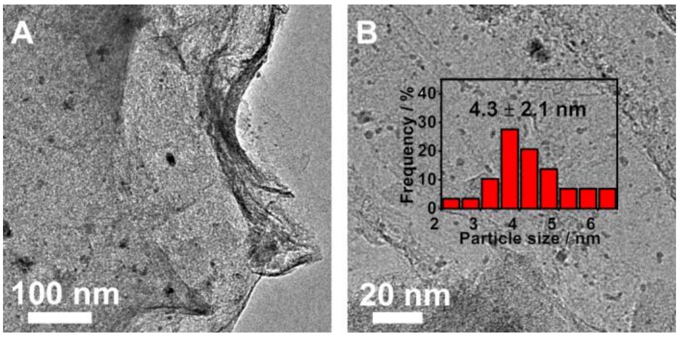

Figure S8. Recovered Rh/LC$_{400}$ after a seven-time recycling. (A-B) TEM images. Insert of (B) is the size distribution histogram by statistical analysis of 200 Rh nanoparticles. Related to Figure 7.

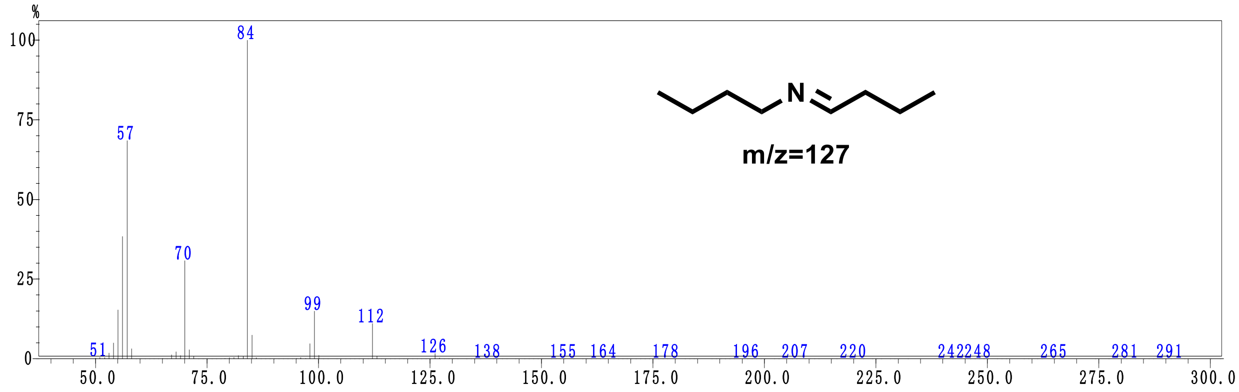

Figure S9. Mass spectrum of *N*-butylbutan-1-imine. Related to Scheme 5.

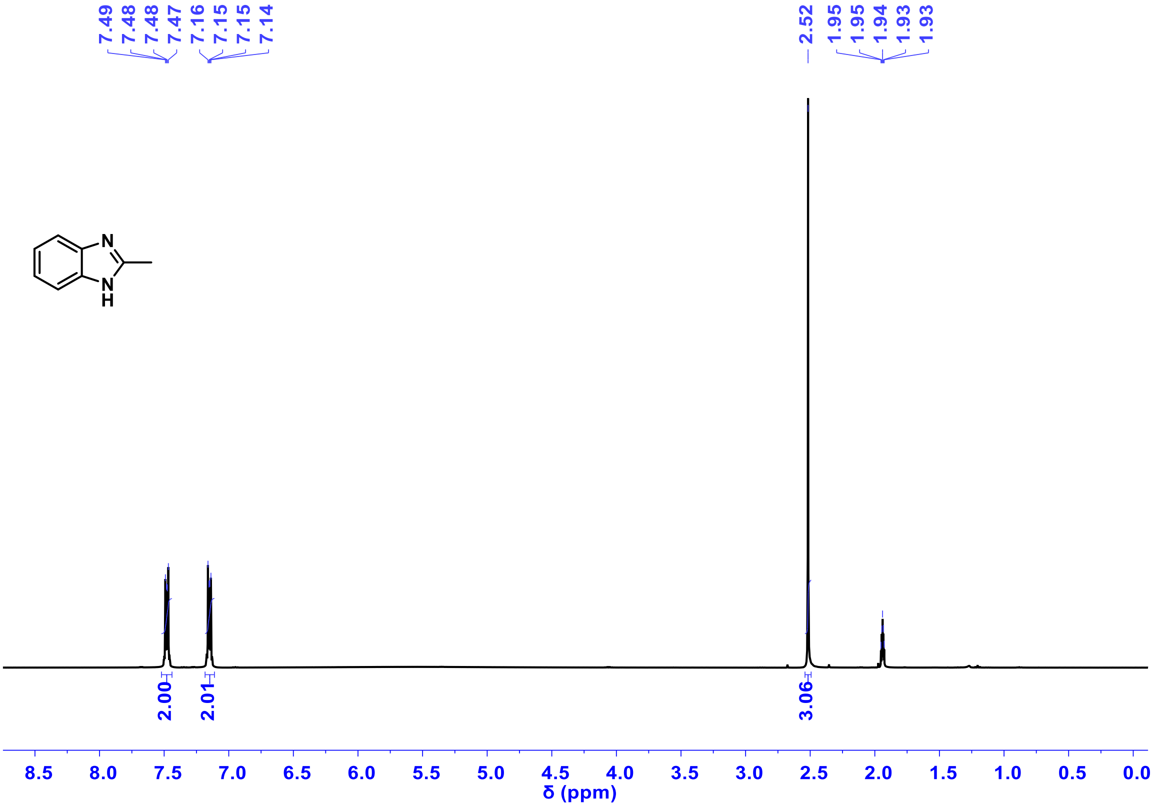

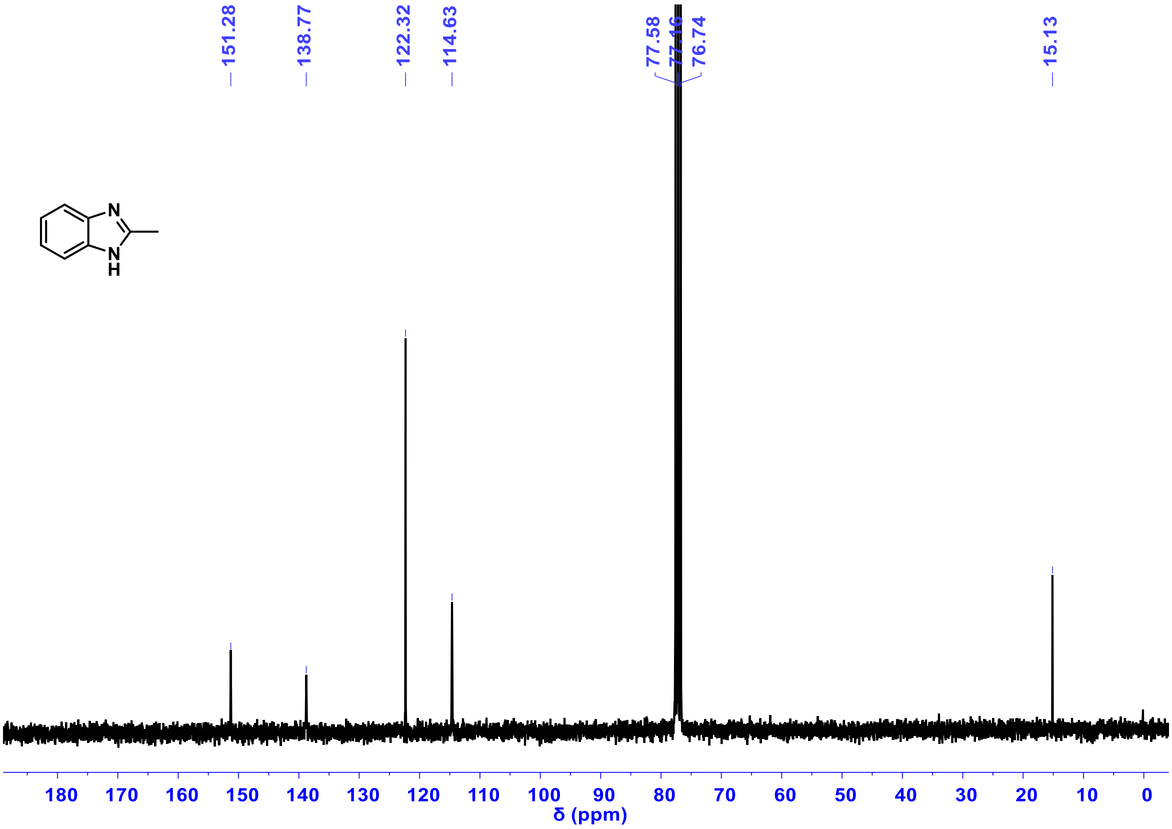

Figure S10. ^{1}H and ^{13}C{^{1}H} NMR of 2-methyl-1*H*-benzo[*d*]imidazole 3a. Related to Figure 8. ^{1}H NMR (300 MHz, CD_3CN, 25°C) δ 7.48 (dd, *J* = 6.0, 3.2 Hz, 2H), 7.15 (dd, *J* = 6.0, 3.2 Hz, 2H), 2.52 (s, 3H). ^{13}C {^{1}H} NMR (75 MHz, $CDCl_3$, 25°C) δ = 151.28, 138.77, 122.32, 114.63, 15.13 ppm.

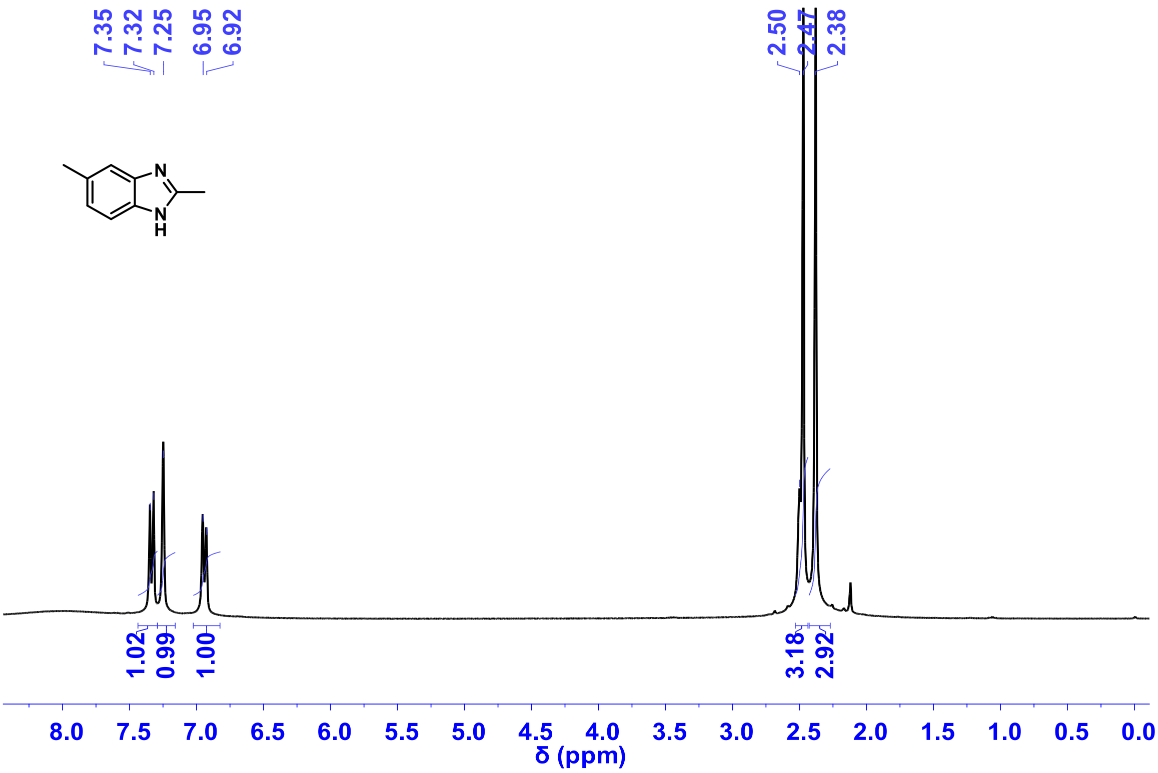

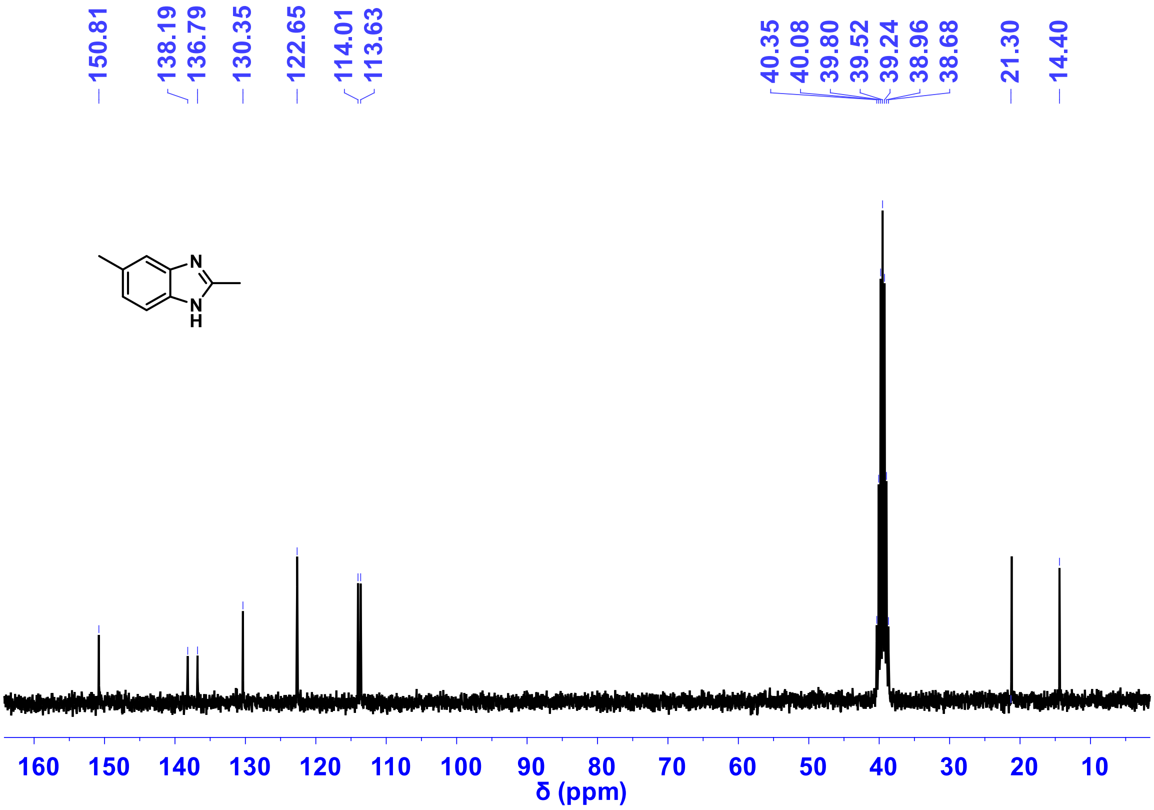

Figure S11. ^{1}H and ^{13}C{^{1}H} NMR of 2,5-dimethyl-1*H*-benzo[*d*]imidazole. Related to Figure 8. ^{1}H NMR (300 MHz, DMSO-d_6, 25 °C) δ 7.35 (d, J = 8.1 Hz, 1H), 7.25 (s, 1H), 6.95 (d, J = 8.1 Hz, 1H), 2.47 (s, 3H), 2.38 (s, 3H). ^{13}C {^{1}H} NMR (75 MHz, DMSO, 25 °C) δ 150.81, 138.19, 136.79, 130.35, 122.65, 114.01, 113.63, 21.30, 14.40.

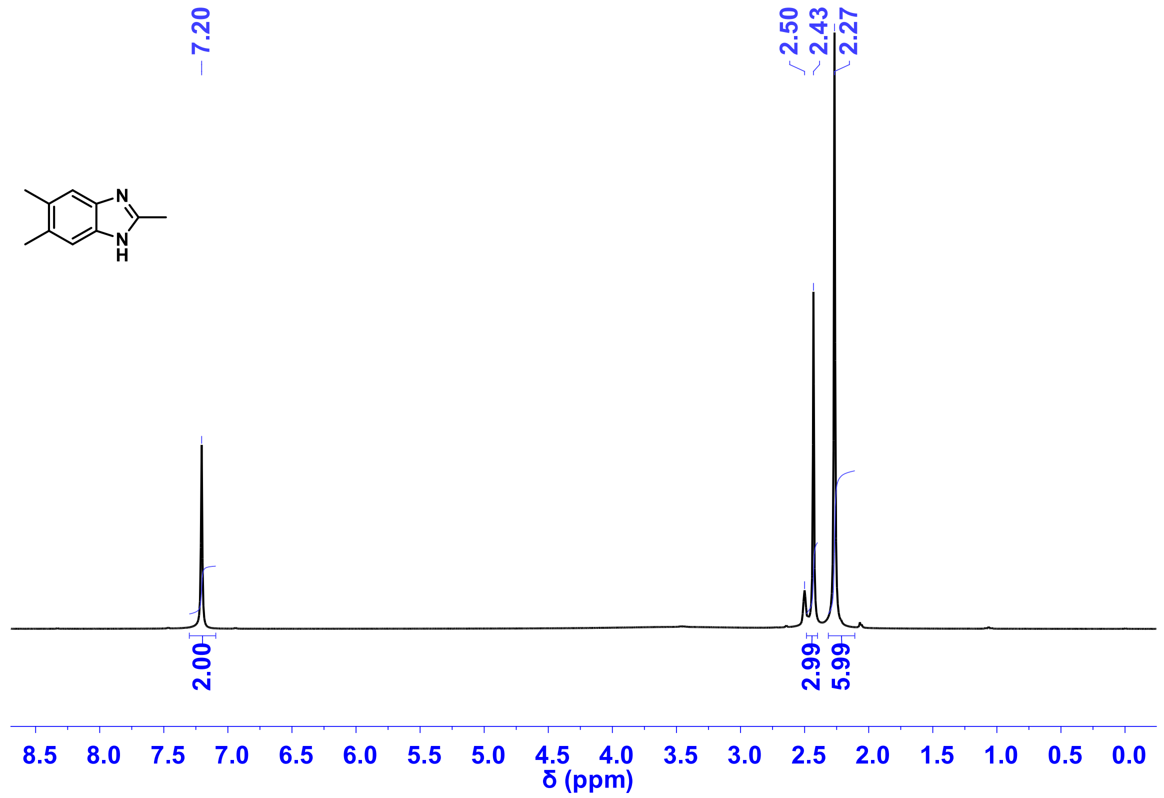

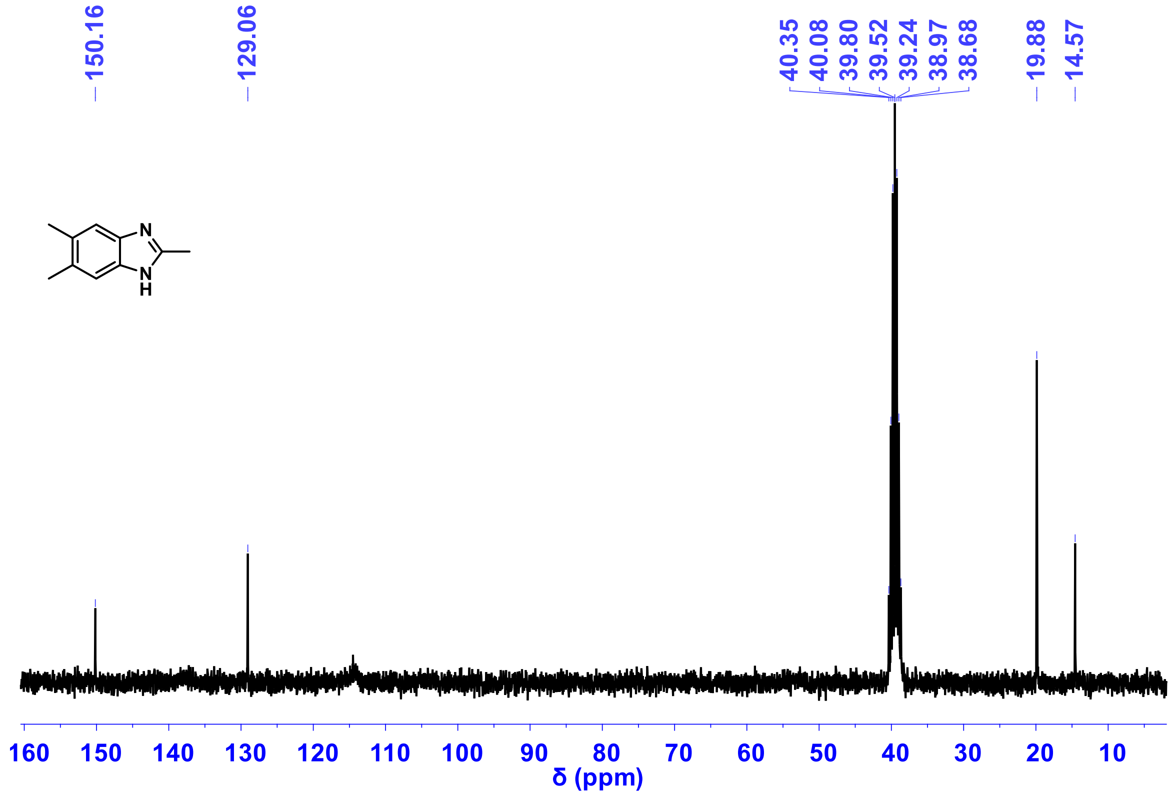

Figure S12. ^{1}H and ^{13}C{^{1}H} NMR of 2,5,6-trimethyl-1*H*-benzo[*d*]imidazole. Related to Figure 8. ^{1}H NMR (300 MHz, DMSO-*d*$_6$, 25 °C) δ 7.20 (s, 2H), 2.43 (s, 3H), 2.27 (s, 6H). ^{13}C {^{1}H} NMR (75 MHz, DMSO-*d*$_6$, 25 °C) δ 150.16, 129.06, 19.88, 14.57.

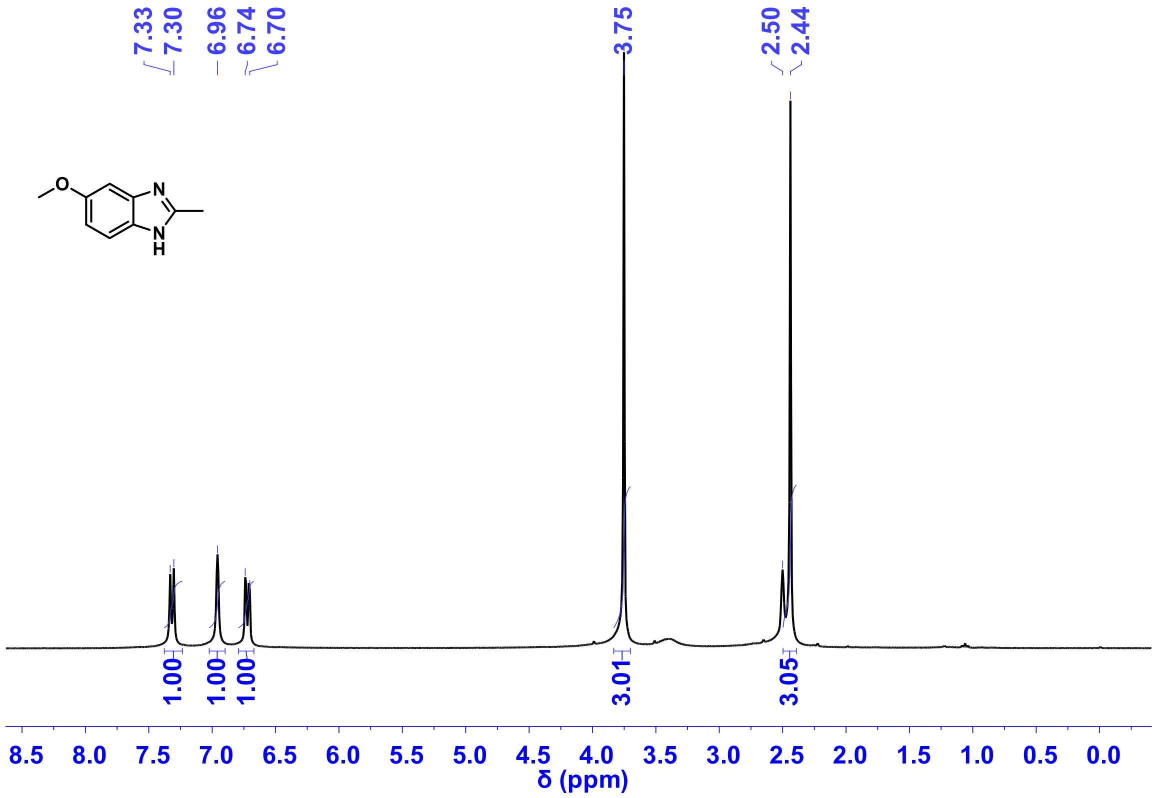

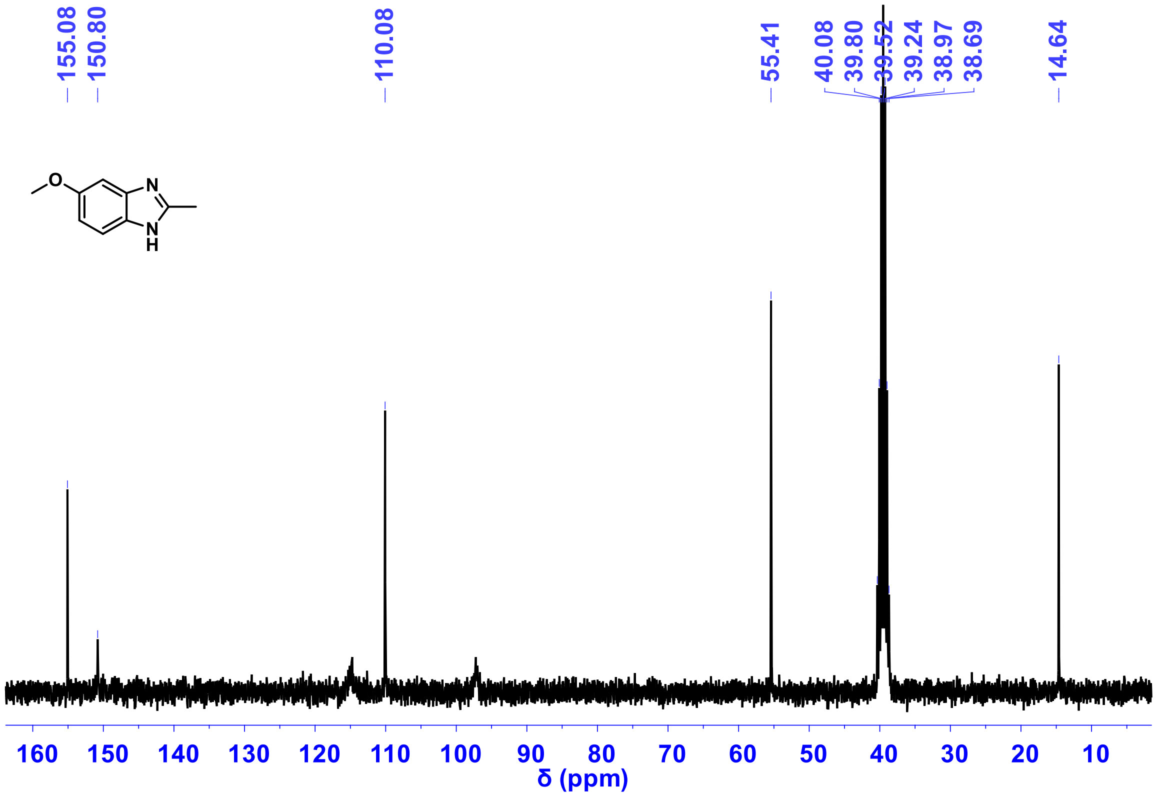

Figure S13. ^{1}H and ^{13}C{^{1}H} NMR of 5-methoxy-2-methyl-1*H*-benzo[*d*]imidazole. Related to Figure 8. ^{1}H NMR (300 MHz, DMSO-d_6, 25°C) δ 7.33 (d, *J* = 8.6 Hz, 1H), 6.96 (s, 1H), 6.74 (d, *J* = 10.7 Hz, 1H), 3.75 (s, 3H), 2.44 (s, 3H). ^{13}C {^{1}H} NMR (75 MHz, DMSO-d_6, 25 °C) δ 155.08, 150.80, 110.08, 55.41, 14.64.

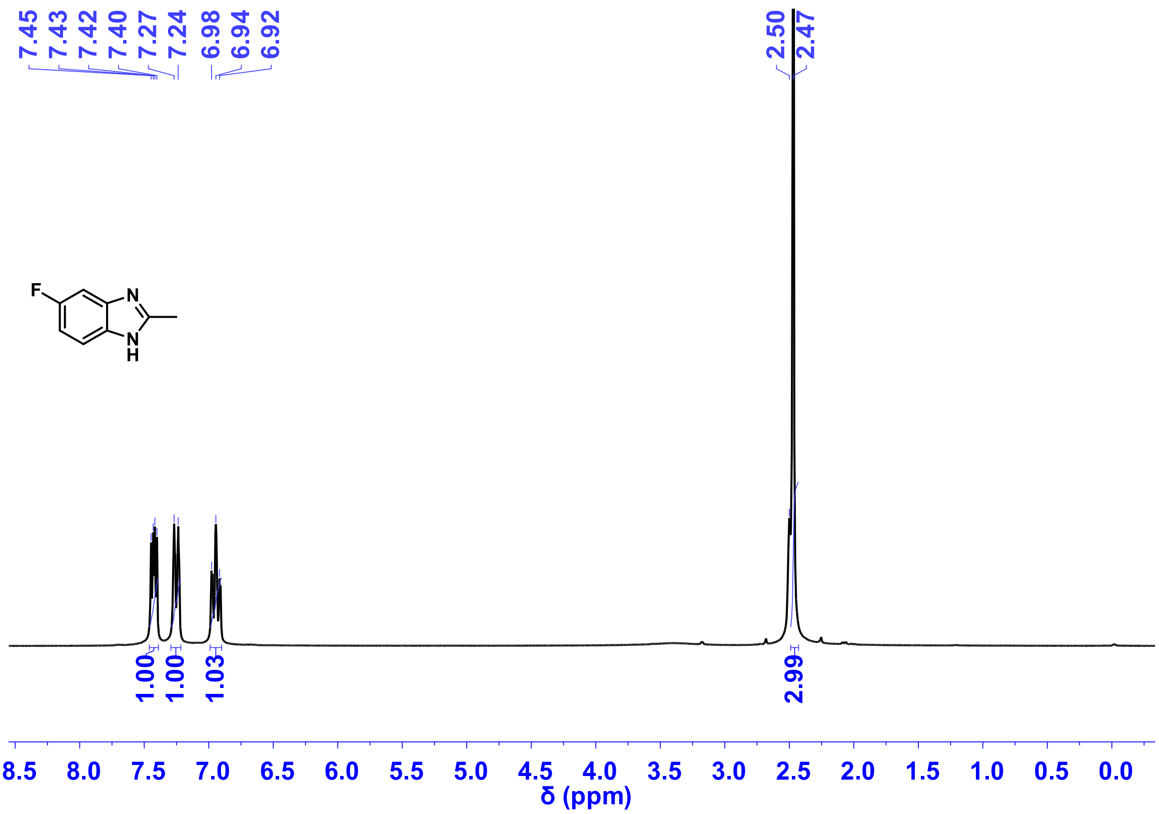

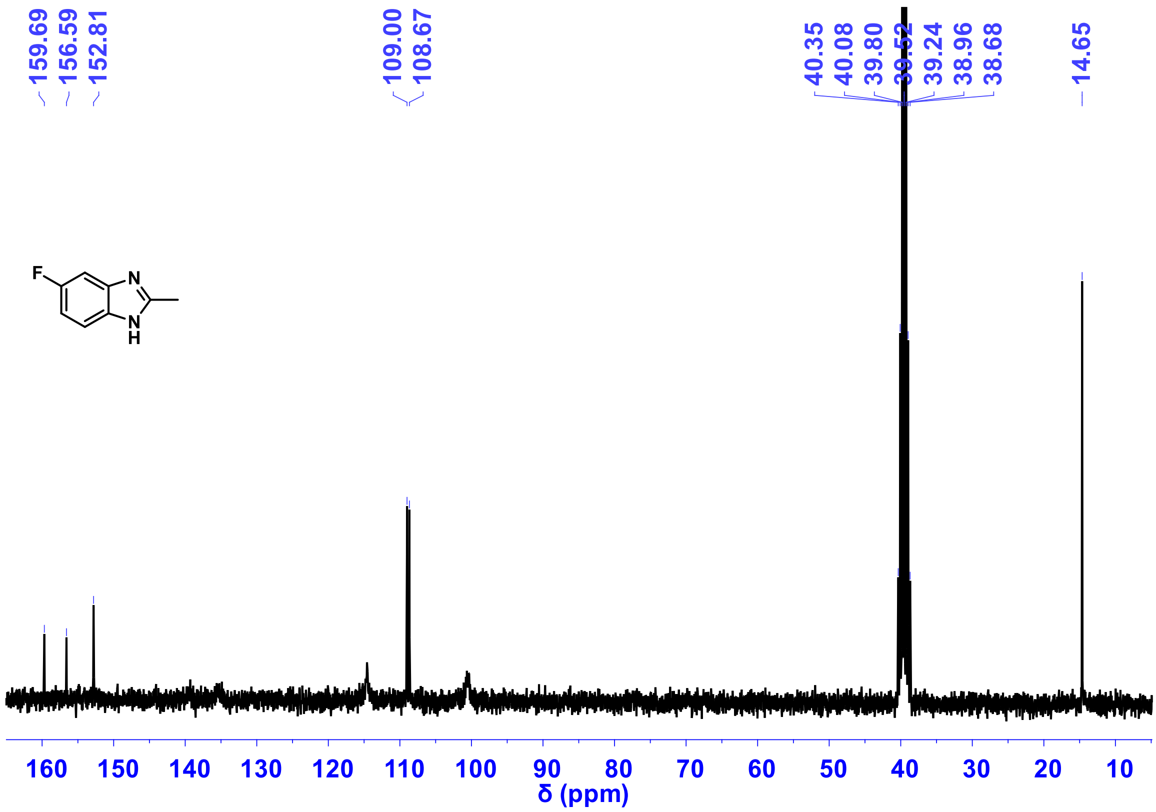

Figure S14. ^{1}H NMR of 5-fluoro-2-methyl-1*H*-benzo[*d*]imidazole. Related to Figure 8. ^{1}H NMR (300 MHz, DMSO-d_6, 25 °C) δ 7.45 (dd, *J* = 8.6, 4.9 Hz, 1H), 7.27 (d, *J* = 9.6 Hz, 1H), 6.98 (t, *J* = 9.3 Hz, 1H), 2.47 (s, 3H). ^{13}C {^{1}H} NMR (75 MHz, DMSO-d_6, 25 °C) δ 159.69, 156.59, 152.81, 109.00, 108.67, 40.80, 14.65.

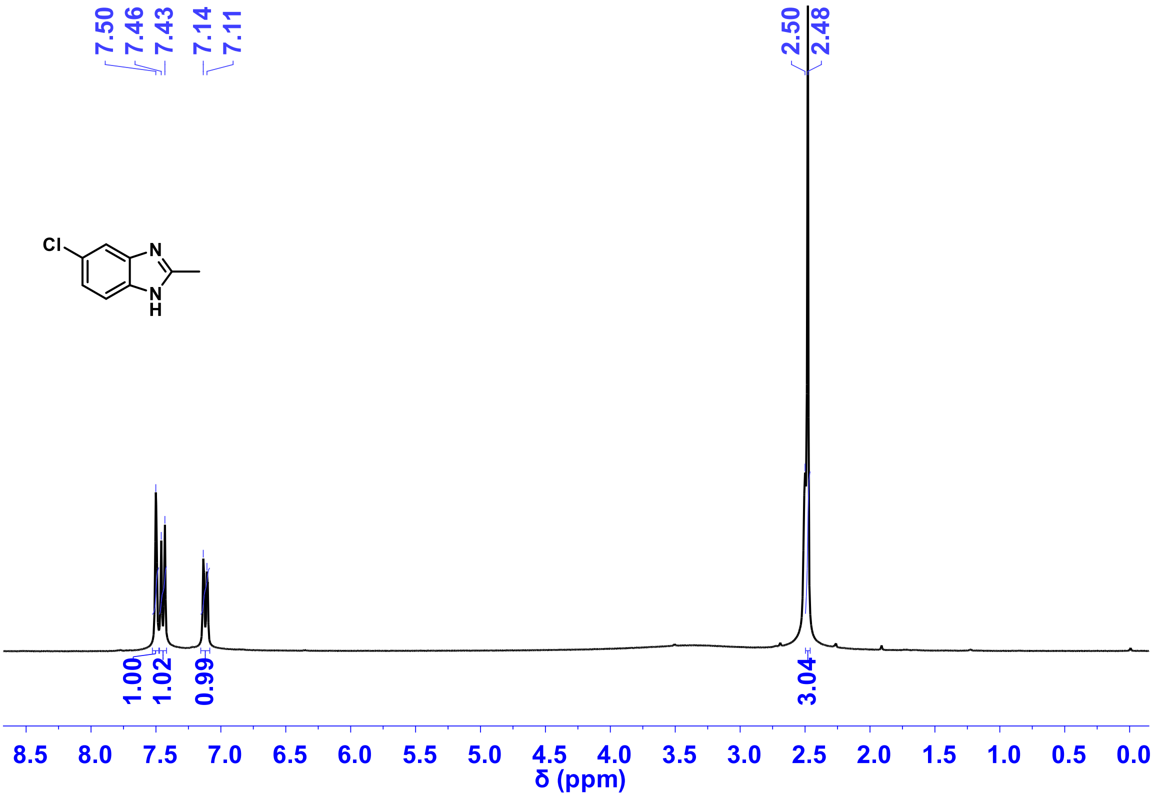

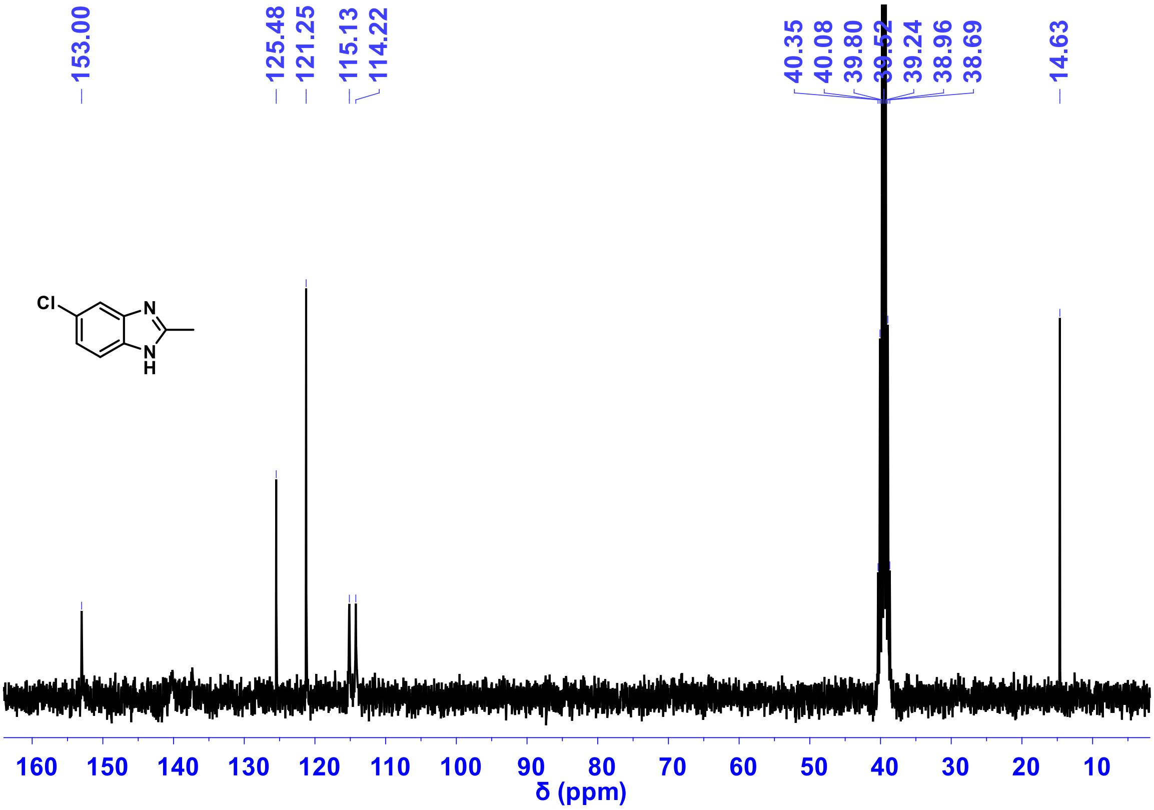

Figure S15. ^{1}H and ^{13}C{^{1}H} NMR of 5-chloro-2-methyl-1*H*-benzo[*d*]imidazole. Related to Figure 8. ^{1}H NMR (300 MHz, DMSO-d_6, 25 °C) δ 7.50 (s, 1H), 7.45 (d, *J* = 8.5 Hz, 1H), 7.14 (d, *J* = 8.5 Hz, 1H), 2.48 (s, 3H). ^{13}C {^{1}H} NMR (75 MHz, DMSO-d_6, 25 °C) δ 153.00, 125.48, 121.25, 115.13, 114.22, 14.63.

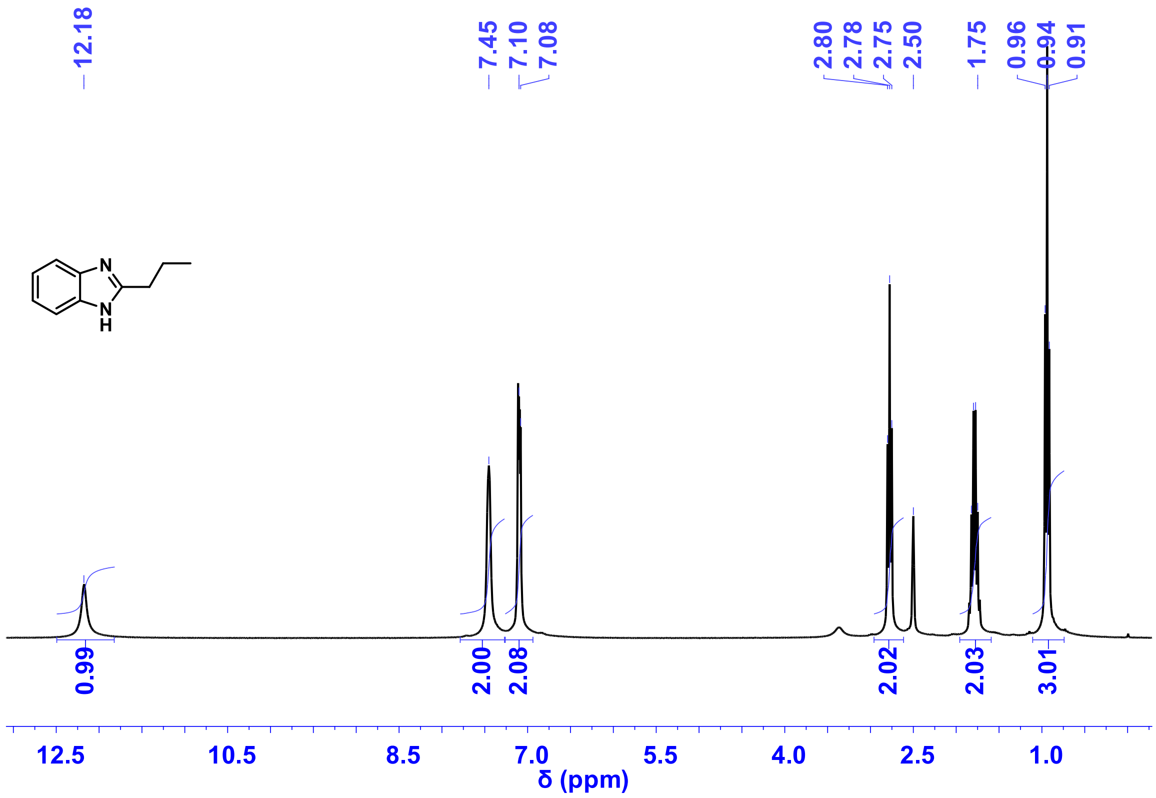

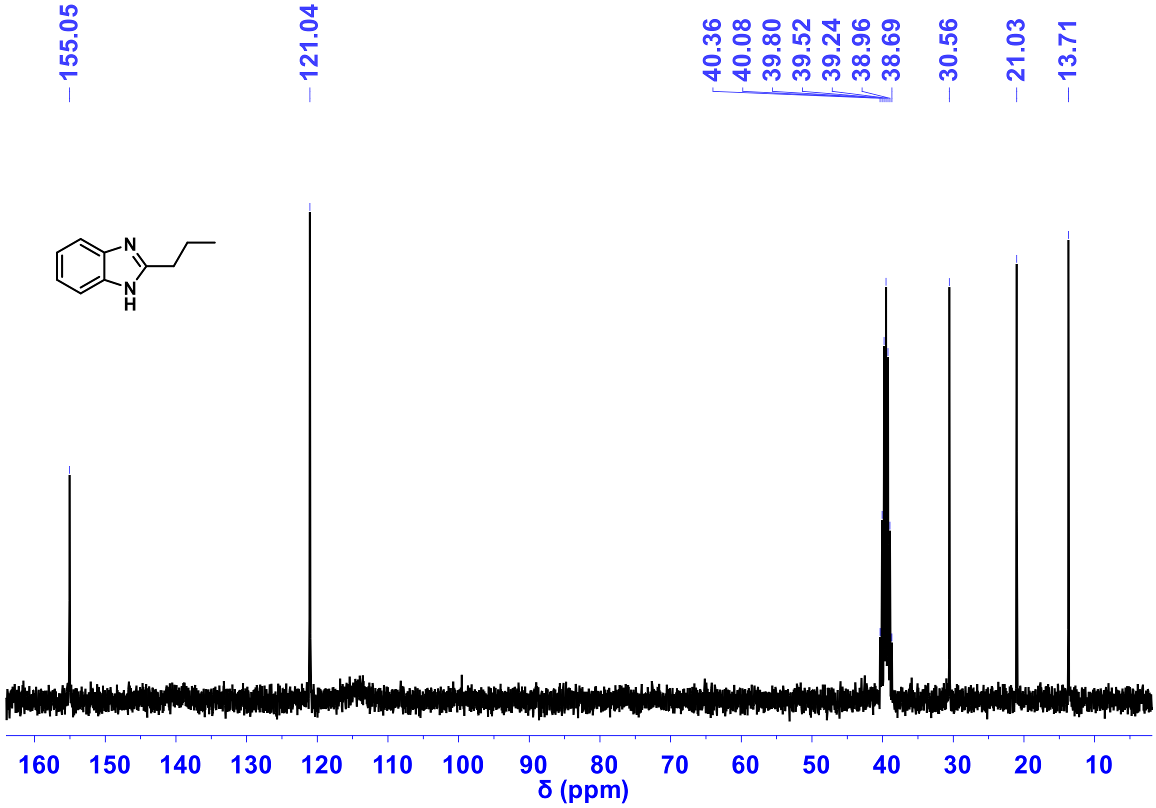

Figure S16. ^{1}H and ^{13}C{^{1}H} NMR of 2-propyl-1*H*-benzo[*d*]imidazole. Related to Figure 8. ^{1}H NMR (300 MHz, DMSO-d_6, 25 °C) δ 12.18 (s, 1H), 7.45 (s, 2H), 7.09 (d, J = 5.9 Hz, 2H), 2.78 (t, J = 7.4 Hz, 2H), 1.75 (dd, J = 14.8, 7.4 Hz, 2H), 0.94 (t, J = 7.4 Hz, 3H). ^{13}C {^{1}H} NMR (75 MHz, DMSO-d_6, 25 °C) δ 155.05, 121.04, 30.56, 21.03, 13.71.

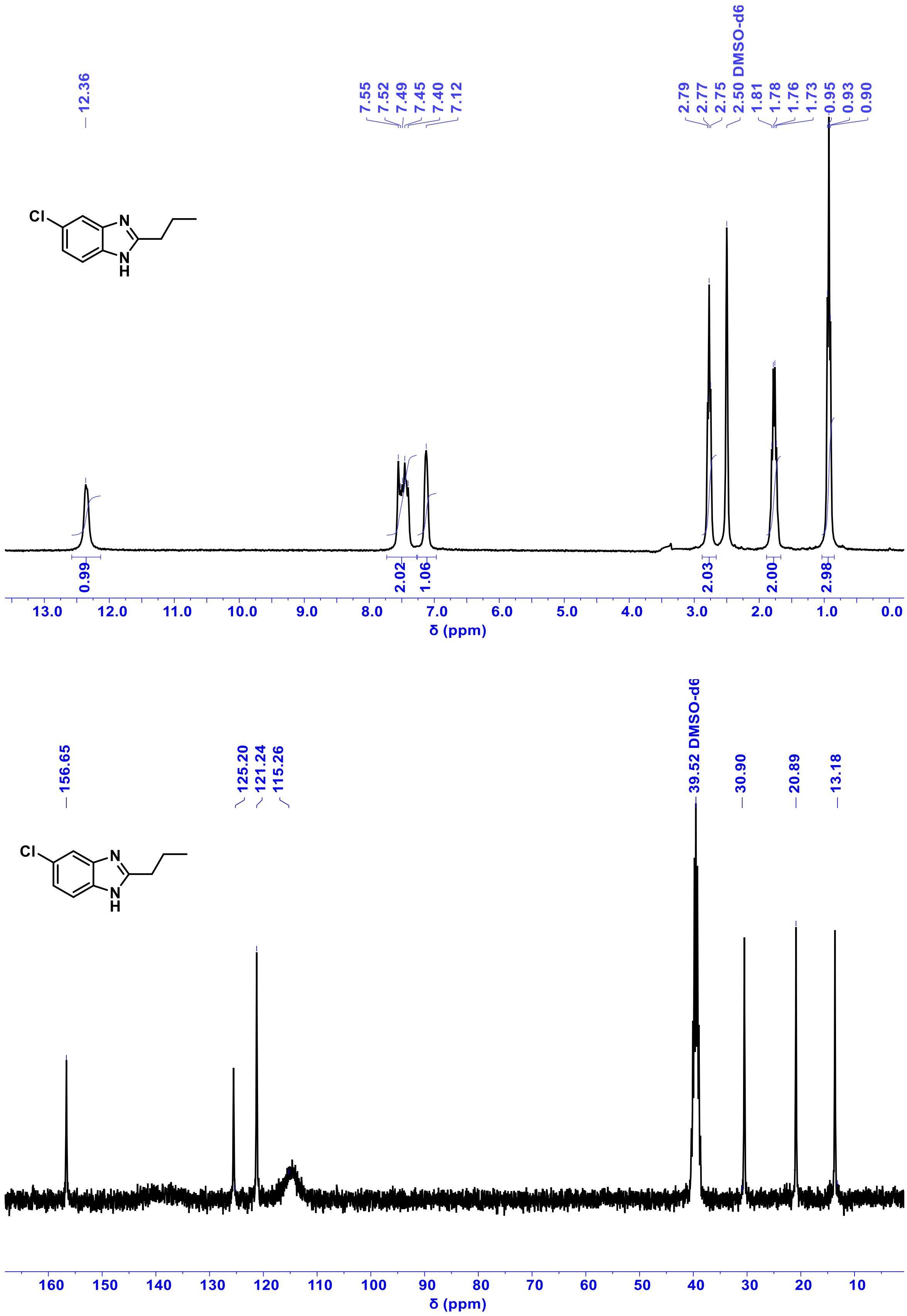

Figure S17. ^{1}H and ^{13}C{^{1}H} NMR of 5-chloro-2-propyl-1*H*-benzo[*d*]imidazole. Related to Figure 8. ^{1}H NMR (300 MHz, DMSO-d_6) δ 12.36 (s, 1H), 7.73 – 7.27 (m, 2H), 7.12 (s, 1H), 2.77 (t, *J* = 7.2 Hz, 2H), 1.77 (q, *J* = 7.3 Hz, 2H), 0.93 (t, *J* = 7.2 Hz, 3H). ^{13}C {^{1}H} NMR (75 MHz, DMSO-d_6, 25 °C) δ 156.65, 125.20, 121.24, 115.26, 30.90, 20.89, 13.18.

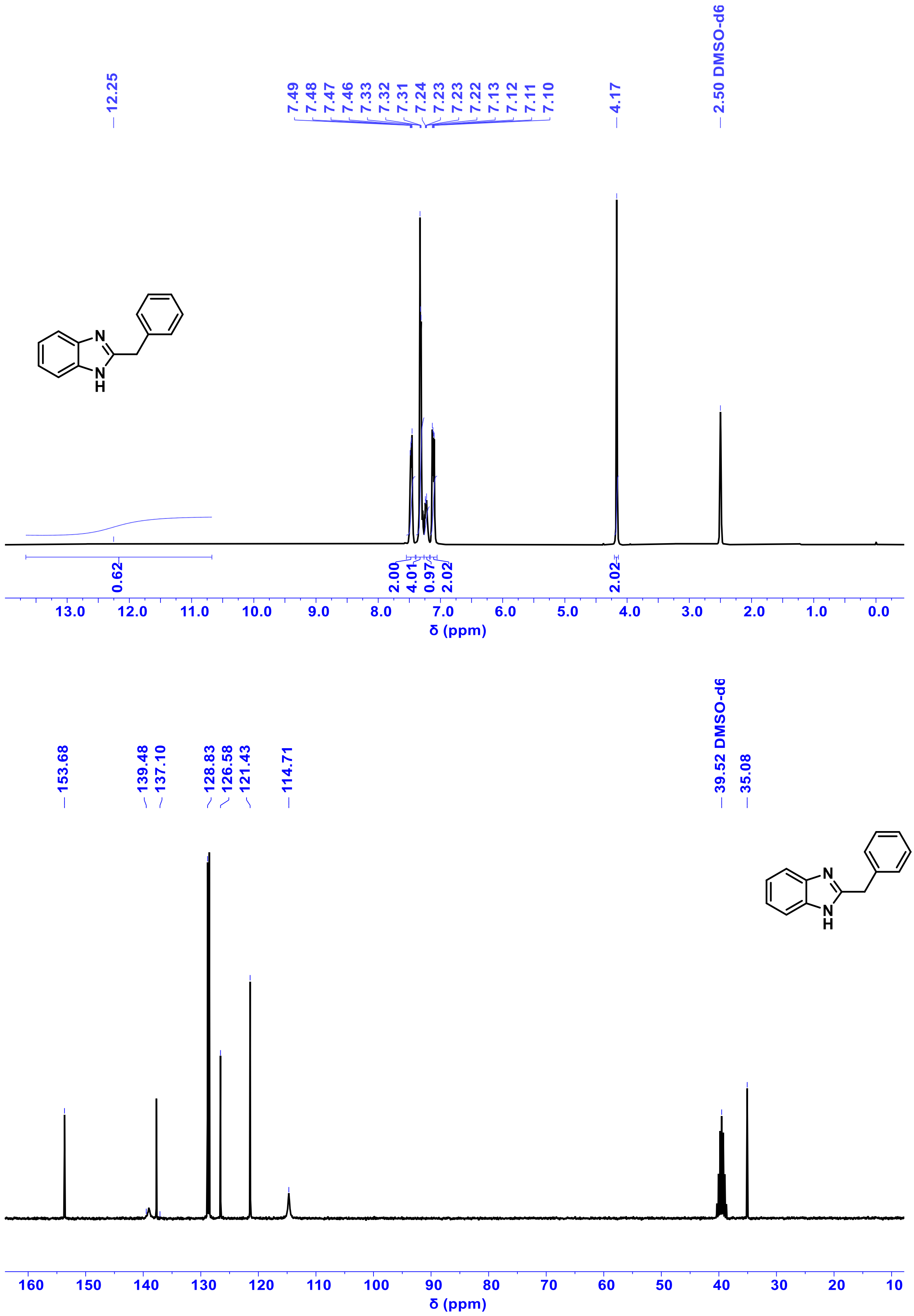

Figure S18. ^{1}H and ^{13}C{^{1}H} NMR of 2-benzyl-1*H*-benzo[*d*]imidazole. Related to Figure 8. ^{1}H NMR (300 MHz, DMSO-d_6) δ 12.25 (s, 1H), 7.47 (dd, *J* = 5.8, 3.2 Hz, 2H), 7.39 – 7.26 (m, 4H), 7.23 (dd, *J* = 3.4, 2.4 Hz, 1H), 7.12 (dd, *J* = 5.9, 3.1 Hz, 2H), 4.17 (s, 2H). ^{13}C{^{1}H} NMR (75 MHz, DMSO-d_6) δ 153.68, 139.48, 137.10, 128.83, 126.58, 121.43, 114.71, 35.08.

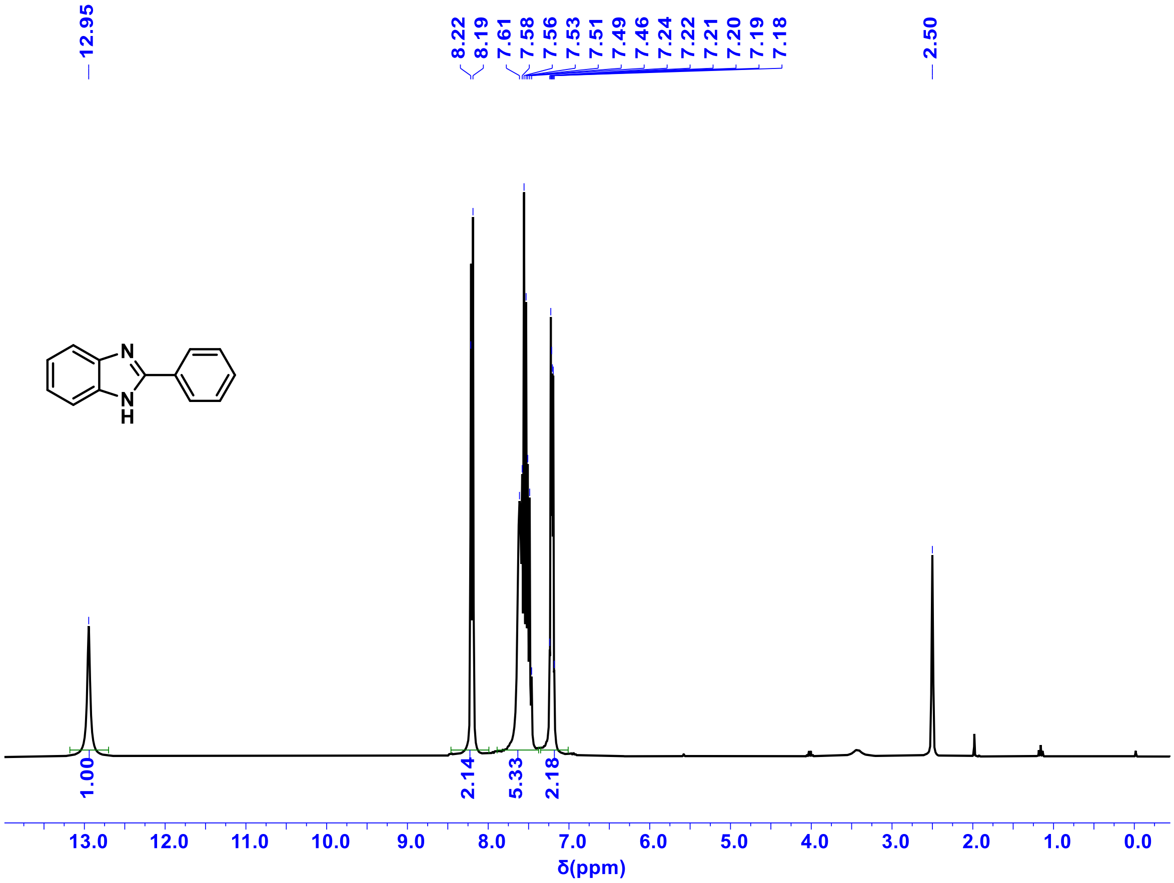

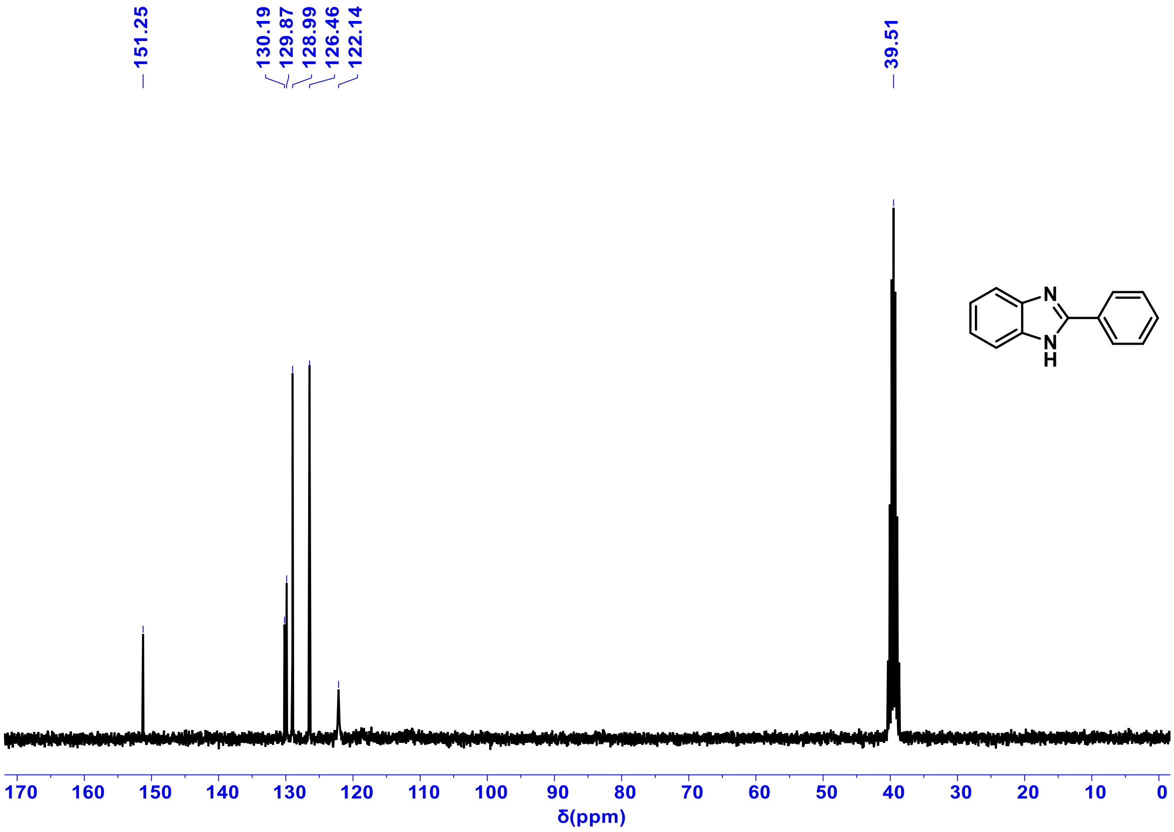

Figure S19. ^{1}H and ^{13}C{^{1}H} NMR of 2-phenyl-1*H*-benzo[*d*]imidazole. Related to Figure 8. ^{1}H NMR (300 MHz, 25 °C, DMSO-*d*6) δ 12.95 (s, 1H), 8.20 (d, *J* = 8.2 Hz, 2H), 7.89 – 7.38 (m, 5H), 7.35 – 7.01 (m, 2H). ^{13}C{^{1}H} NMR (75 MHz, 25 °C, DMSO-*d*6) δ 151.25, 130.19, 129.87, 128.99, 126.46, 122.14.

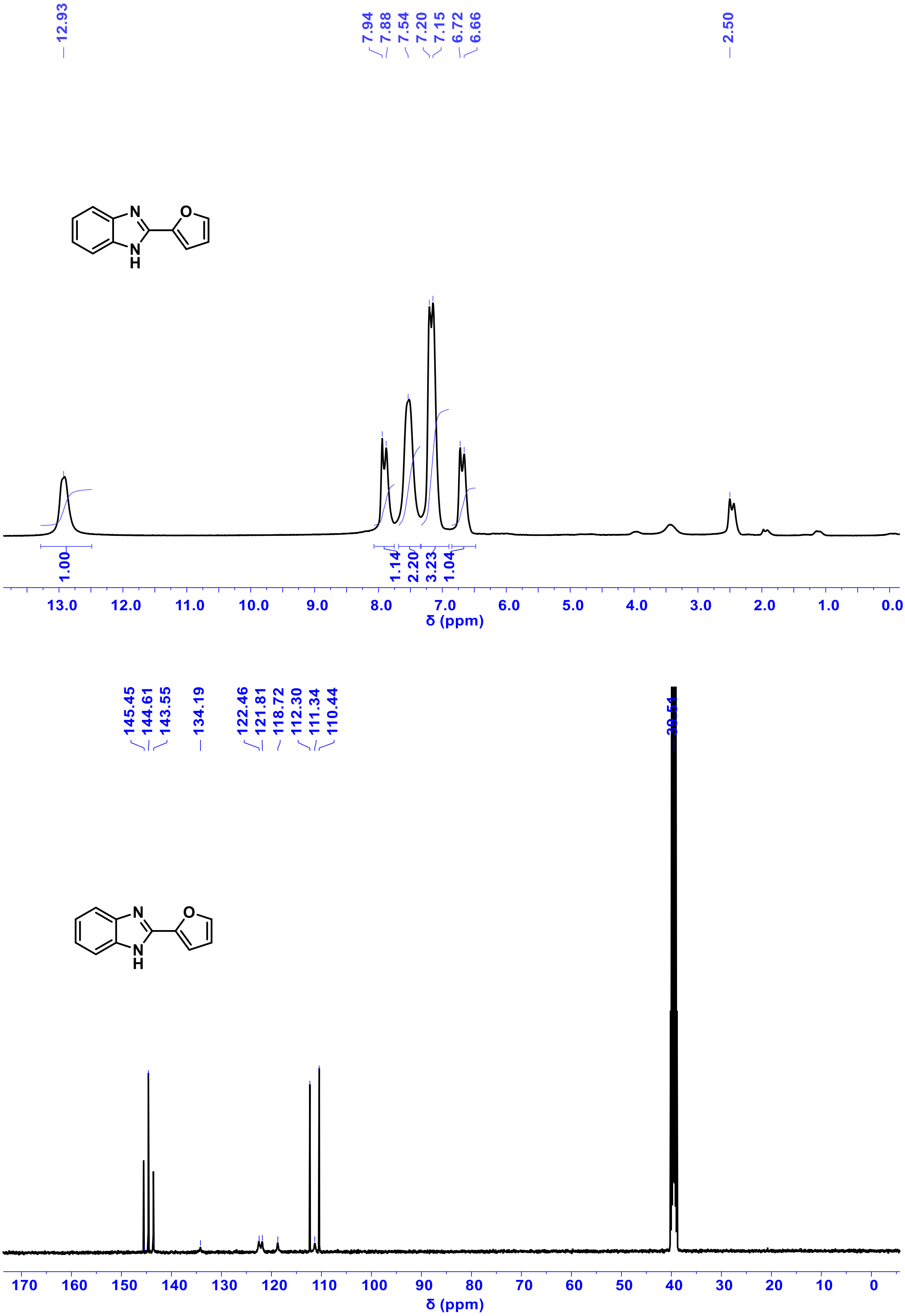

Figure S20. ^{1}H and ^{13}C{^{1}H} NMR of 2-(2-furanyl)-1*H*-benzo[*d*]imidazole. Related to Figure 8. ^{1}H NMR (300 MHz, 25 °C, DMSO-*d*6) δ 12.93 (s, 1H), 7.91 (d, *J* = 18.7 Hz, 1H), 7.54 (s, 2H), 7.18 (d, *J* = 16.8 Hz, 3H), 6.69 (d, *J* = 19.4 Hz, 1H). ^{13}C{^{1}H} NMR (101 MHz, 25 °C, DMSO-*d*6) δ 145.45, 144.61, 143.55, 134.19, 122.46, 121.81, 118.72, 112.30, 111.34, 110.44.
